# Catalytic asymmetric synthesis of biologically important 3-hydroxyoxindoles: an update

**DOI:** 10.3762/bjoc.12.98

**Published:** 2016-05-18

**Authors:** Bin Yu, Hui Xing, De-Quan Yu, Hong-Min Liu

**Affiliations:** 1 School of Pharmaceutical Sciences & Collaborative Innovation Center of New Drug Research and Safety Evaluation, Zhengzhou University, Zhengzhou 450001, China; 2 School of Chemistry and Molecular Biosciences, University of Queensland, Brisbane, 4072, Queensland, Australia

**Keywords:** 3-hydroxyoxindoles, oxindoles, organocatalysis, spirooxindoles, transition metal catalysis

## Abstract

Oxindole scaffolds are prevalent in natural products and have been recognized as privileged substructures in new drug discovery. Several oxindole-containing compounds have advanced into clinical trials for the treatment of different diseases. Among these compounds, enantioenriched 3-hydroxyoxindole scaffolds also exist in natural products and have proven to possess promising biological activities. A large number of catalytic asymmetric strategies toward the construction of 3-hydroxyoxindoles based on transition metal catalysis and organocatalysis have been reported in the last decades. Additionally, 3-hydroxyoxindoles as versatile precursors have also been used in the total synthesis of natural products and for constructing structurally novel scaffolds. In this review, we aim to provide an overview about the catalytic asymmetric synthesis of biologically important 3-substituted 3-hydroxyoxindoles and 3-hydroxyoxindole-based further transformations.

## Introduction

**Figure 1:**
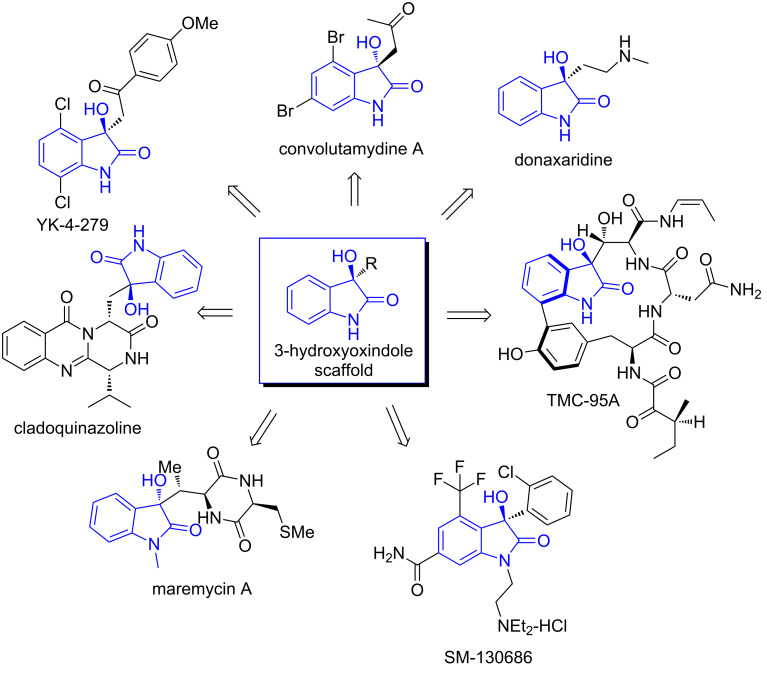
3-Hydroxyoxindole-containing natural products and biologically active molecules.

Chiral oxindoles are an important class of compounds, which widely exist in nature and have exhibited diverse biological activities [1–7]. Of particular interest are optically active 3-hydroxyoxindoles (also known as 3-hydroxyindolin-2-one and 3-hydroxy-2-oxindole), which are also prevalent in natural products and biologically important molecules (Figure 1). 3-Hydroxyoxindole-containing derivatives have recently drawn extensive attention due to their diverse biological activities [8]. Several 3-hydroxyoxindole-derived compounds are undergoing preclinical evaluation. For example, the well-known natural product TMC-95A is able to inhibit proteasome non-covalently and reversibly [9]. SM-130686 is currently being used for the treatment of growth hormone deficiency as a potent and orally active GHSR agonist [10]. YK-4-279 can potently inhibit the growth of Ewing’s sarcoma by blocking the interaction between the oncogenic protein EWS-FLI1 and RNA helicase A (RHA) [11]. Interestingly, only (*S*)-YK-4-279 has been reported to be able to inhibit the EWS-FLI1/RHA interactions specifically, significantly more potent than its (*R*)-enantiomer and racemic compound [12]. Additionally, the 3-hydroxyoxindoles as versatile intermediates have also been used to construct small-molecule libraries for drug screening.

Inspired by the biological potential and synthetic utility, metal- or organo-catalyzed asymmetric synthesis of chiral 3-hydroxyoxindoles have been highly pursued in the last decades. An excellent review by Chimni and co-workers summarized the catalytic strategies for the enantioselective synthesis of chiral 3-hydroxyoxindoles [13]. During the last three years, significant progress on the catalytic asymmetric synthesis of enantioenriched 3-hydroxyoxindoles has been observed. In this review, we aim to provide an update about the asymmetric synthesis of 3-hydroxyoxindoles, literatures from 2013 to 2016 are covered. Besides, recent progress on the 3-hydroxyoxindole-based further transformations are covered in this review. Perspectives and future directions are also discussed based on previous reports and our own understandings. This review is organized based on the catalyst types.

## Review

### Transition metal-catalyzed synthesis

The chiral ligand/metal complexes have been widely employed in catalytic asymmetric synthesis of enantioenriched 3-hydroxyoxindoles, achieving good to excellent enantioselectivities and high yields.

#### Pd-catalyzed allylation of isatins

**Scheme 1:**
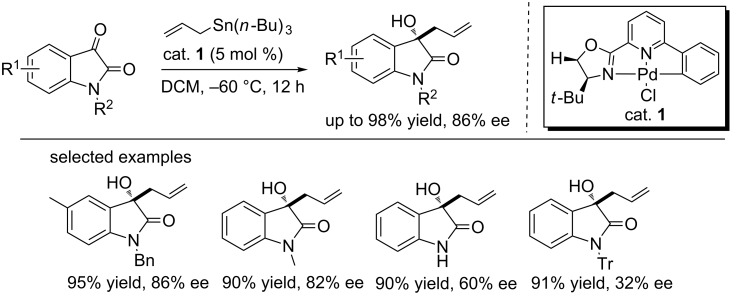
Chiral CNN pincer Pd(II) complex **1** catalyzed asymmetric allylation of isatins.

**Scheme 2:**
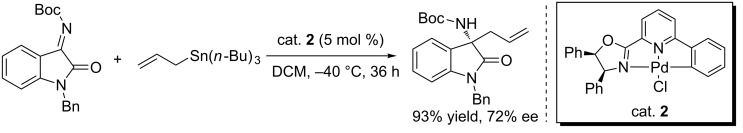
Asymmetric allylation of ketimine catalyzed by the chiral CNN pincer Pd(II) complex **2**.

The palladium catalyst is widely used in organic synthesis and has showed its usefulness in the allylation of isatins. In 2014, Song and co-workers designed the chiral CNN (three atoms attched to the palladium) pincer Pd complexes, which were proved to be efficient in catalyzing the enantioselective allylation of isatins with allyltributytin (Scheme 1) [14]. 3-Allyl-3-hydroxyoxindoles were obtained in 89–98% yield and with 32–86% ee when the reactions were carried out at −60 °C using 5 mol % of CNN pincer Pd complex (cat. **1**). Substituents attached to the isatin aromatic ring and *N*-protecting groups were important in controlling the stereoselectivity. Products bearing electron-donating groups on the isatin core were obtained with high enantioselectivities. When the R^2^ group was 1-naphthylmethyl, trityl (Tr) or hydrogen, low ee values were observed. This protocol was successfully applied to the synthesis of 3-aminooxindoles, affording the allylated product in 93% yield and 72% ee value when cat. **2** was used (Scheme 2).

**Scheme 3:**
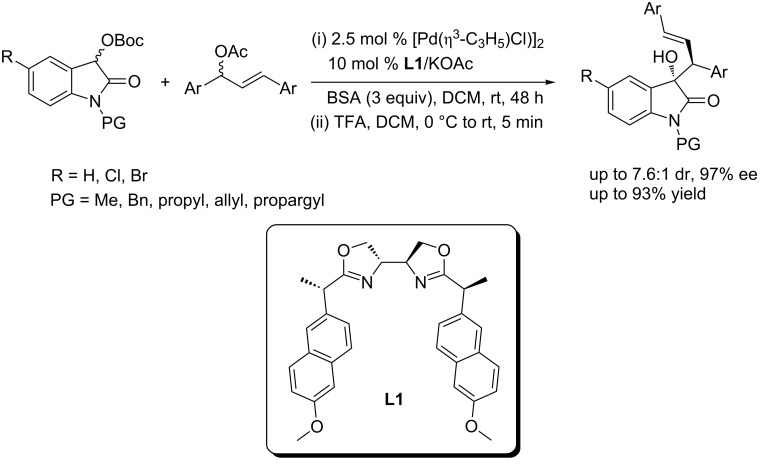
Pd/**L1** complex-catalyzed asymmetric allylation of 3-*O-*Boc-oxindoles.

Kesavan and co-workers described that the Pd/bis(oxazoline) (**L1**) complex can catalyze the asymmetric allylation of 3-*O-*Boc-oxindole, yielding the 3-allyl-3-hydroxyoxindoles in good yields (up to 93% yield) and with high enantioselectivities (up to 97% ee) and diastereoselectivities (up to 7.6:1 dr, Scheme 3) [15]. The best condition was 2.5 mol % of [Pd(η^3^-C_3_H_5_)Cl)]_2_, 10 mol % of **L1** as the ligand and 10 mol % of KOAc as an additive, and 3 equiv of BSA (*N,O*-bis(trimethylsilyl)acetamide). A broad substrate scope and functional group tolerance were observed. However, no reaction occurred between the *ortho*-chloro substituted 1,3-diaryl-2-propenyl acetate and 3-*O-*Boc-oxindole because the chlorine atom inhibited the formation of the active π-allyl-palladium species.

#### Cu-catalyzed direct addition

**Scheme 4:**
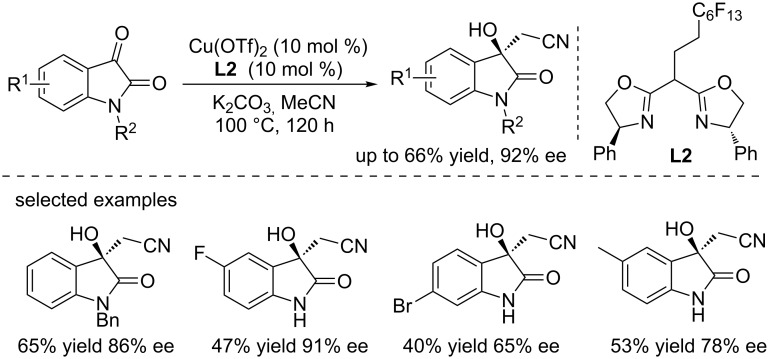
Cu(OTf)_2_-catalyzed asymmetric direct addition of acetonitrile to isatins.

The copper catalysts have gained an increasing attention in the last decades and have been successfully employed in various transformations. In 2014, Cai and co-workers reported a copper-catalyzed asymmetric direct addition of acetonitrile to isatins in the presence of K_2_CO_3_ (Scheme 4) [16]. The reactions were conducted in acetonitrile using the Cu(OTf)_2_ as the catalyst (10 mol %) and a fluorinated bis(oxazoline) **L2** as the ligand (10 mol %), affording the products in moderate yields (up to 66% yield) and with good to excellent enantioselectivities (up to 92% ee). Interestingly, the fluorinated bis(oxazoline) **L2** can be recovered and reused for a few times without activity loss. However, a long reaction time (120 h) was needed to achieve good results, and the low yields for *N*-unprotected substrates could be explained by the fact that the amide NH (p*K*_a_ in DMSO ≈18.5) is more prone to be activated by K_2_CO_3_ than MeCN (p*K*_a_ = 25). The long reaction times may cause the decomposition of products in the presence of K_2_CO_3_.

**Scheme 5:**
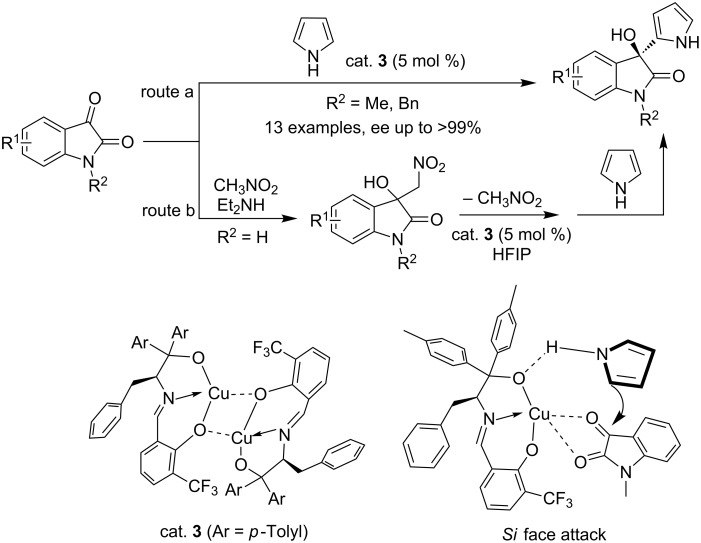
Chiral tridentate Schiff base/Cu complex catalyzed asymmetric Friedel–Crafts alkylation of isatins with pyrrole.

In 2014, Wang et al. designed the chiral tridentate Schiff-base/Cu complex (cat. **3**) for the asymmetric Friedel–Crafts alkylation of pyrrole with isatins (route a, Scheme 5) [17]. For *N*-protected isatin substrates, the final compounds were obtained in excellent enantioselectivities (up to >99% ee), while only moderate enantioselectivities (56–69% ee) were obtained for *N*-unprotected isatin substrates. To address this problem, a modified strategy (route b, Scheme 5) was then applied by controlling the concentration of isatin in situ, giving the corresponding products with 93–99% ee. This modified protocol was also extended to electron-rich heteroarene substrates. Interestingly, hexafluoroisopropanol (HFIP) was found to be able to improve the enantioselectivity significantly. The authors also proposed a possible transition state for the observed stereoselectivity. Pyrrole attacked the ketone from the *Si* face to generate (*R*)-3-pyrrolyl-3-hydroxyoxindoles.

**Scheme 6:**
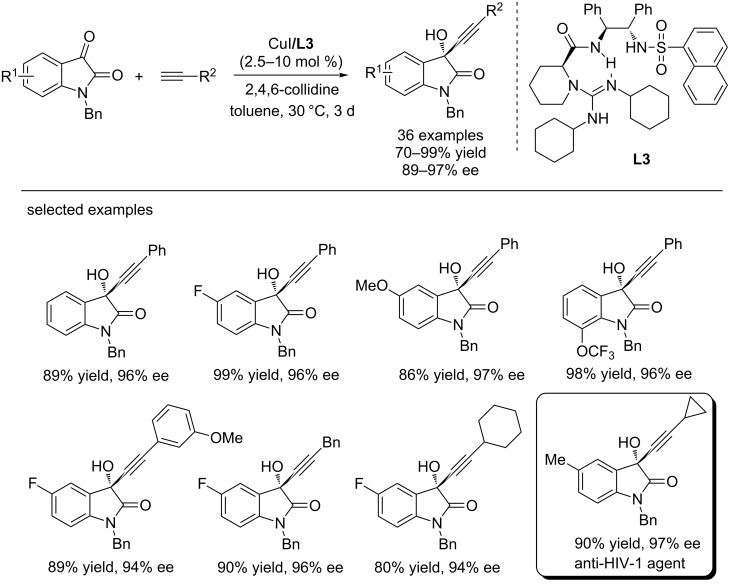
Guanidine/CuI-catalyzed asymmetric alkynylation of isatins with terminal alkynes.

Very recently, Feng et al. revealed that the bifunctional guanidine (**L3**)/CuI catalyst can catalyze asymmetric alkynylation of isatins with terminal alkynes, affording biologically important 3-alkynylated 3-hydroxyoxindoles with good levels of reactivity (up to 99% yield) and excellent enantioselectivities (up to 97% ee, Scheme 6) [18]. The reactions showed a broad substrate scope, regradless of their electronic properties and positions. The protocol was also successfully applied to the synthesis of a drug candidate, which had been proved to possess better inhibitory activity than efavirenz against HIV-1 reverse transcriptase [19]. Besides, the reactions may provide an efficient route to the optically pure propargylic alcohols.

#### Ir-catalyzed synthesis of 3-hydroxyoxindoles

**Scheme 7:**
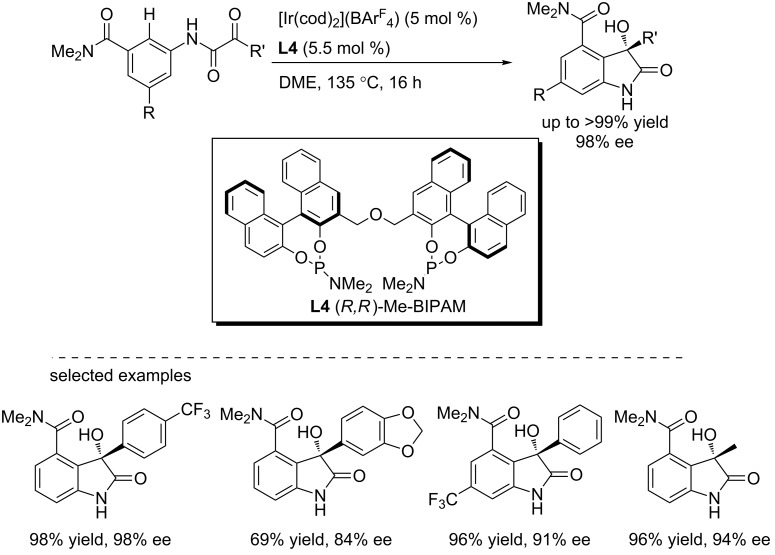
Asymmetric intramolecular direct hydroarylation of α-ketoamides.

Recently, iridium has emerged as a powerful catalyst for C–H bond functionalization [20–23]. In 2014, Yamamoto and co-workers reported a cationic iridium complex catalyzed asymmetric intramolecular hydroarylation of α-ketoamides, yielding 3-substituted 3-hydroxy-2-oxindoles with high regioselectivities and enantioselectivities (up to 98% ee, Scheme 7) [24]. The reactions were performed in 1,2-dimethoxyethane using 5 mol % of [Ir(cod)_2_](BAr^F^_4_) as the catalyst and 5.5 mol % of the chiral ligand **L4**. A wide range of aromatic and aliphatic α-ketoamides were tolerated under the optimized conditions.

**Scheme 8:**
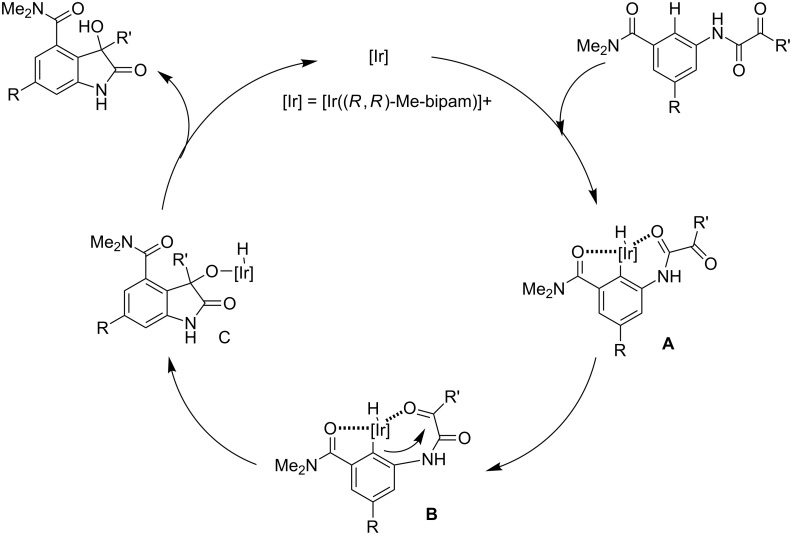
Plausible catalytic cycle for the direct hydroarylation of *α*-ketoamides.

A plausible catalytic cycle was proposed as shown in Scheme 8: [Ir(cod)_2_](BAr^F^_4_) and the ligand **L4** formed the [Ir] precatalyst in situ, which then activated a C–H bond of the substrate to generate aryl–iridium complex **A**. Subsequent intramolecular asymmetric hydroarylation of intermediate **B** produced iridium alkoxide species **C**. Reductive elimination of species **C** gave the product and regenerated the active iridium catalyst.

**Scheme 9:**
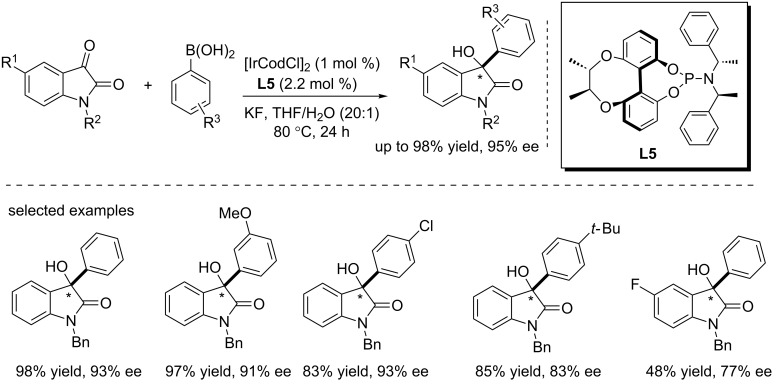
Ir-catalyzed asymmetric arylation of isatins with arylboronic acids.

Recently, Qiu and co-workers developed a novel chiral ligand **L5** based on a chiral-bridged biphenyl backbone and successfully achieved the asymmetric addition of arylboronic acids to *N*-protected isatins using the Ir/**L5** complex, giving the products in high yields (up to 98%) and with good to excellent enantioselectivities (up to 95% ee, Scheme 9) [25]. Only 1 mol % catalyst and 2.2 mol % ligand were used in the reactions. Substituents attached to the aromatic rings of isatins and arylboronic acids, as well as the rigidity of the ligand had remarkable influence on the yields and stereoselectivity. Increase of the rigidity can improve the yields and stereoselectivity.

### Other metal-catalyzed syntheses of 3-hydroxyoxindoles

**Scheme 10:**
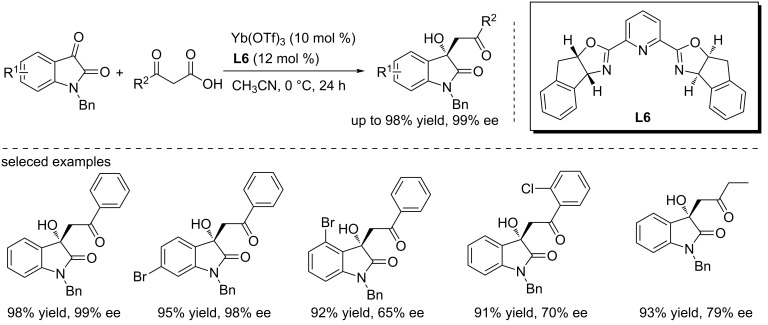
Enantioselective decarboxylative addition of β-ketoacids to isatins.

Aside from above mentioned metal catalysts, several other metal catalysts have also been used for the synthesis of chiral 3-hydroxyoxindoles. In 2013, Pan and co-workers reported the Yb(OTf)_3_-catalyzed enantioselective decarboxylative addition of β-ketoacids to isatins, forming the 3-hydroxyoxindoles in excellent yields (up to 98% yield) and with high enantioselectivities (up to 99% ee, Scheme 10) [26]. The reaction involved a decarboxylation and an Yb(OTf)_3_/PyBox (**L6**)-catalyzed aldol addition. 4 Å Molecular sieves were found to be able to enhance the stereoselectivity. Steric hindrance between substituents and the metal/ligand complex slightly decreased the ee values. In addition, aliphatic β-ketoacids can also be used in the reaction, giving the corresponding alkylated 3-hydroxyoxindoles in good results under the optimized conditions.

**Scheme 11:**
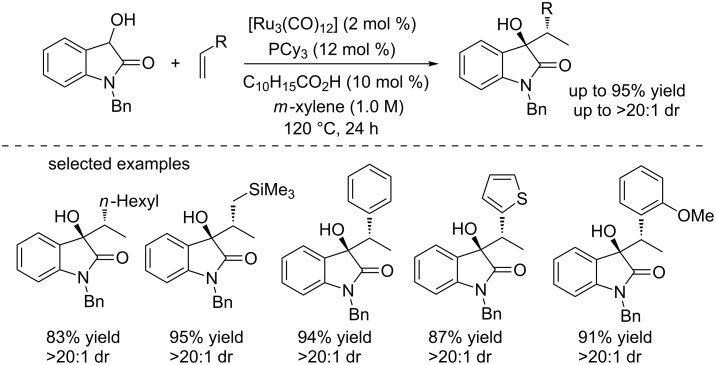
Ruthenium-catalyzed hydrohydroxyalkylation of olefins and 3-hydroxy-2-oxindoles.

Krische and co-workers reported the first Ru-catalyzed hydrohydroxyalkylation of unactivated olefins. The direct C–C coupling reactions of α-olefins and styrenes with 3-hydroxy-2-oxindoles were achieved by using the ruthenium complex, giving the products as single diastereoisomers (Scheme 11) [27]. Interestingly, 1-adamantanecarboxylic acid (10 mol %) as a co-catalyst can remarkably enhance the yields from trace to >90%. A broad range of substituted olefins and unprotected 3-hydroxy-2-oxindoles were examined under these conditions, giving the corresponding products in excellent yields (up to 95%) and with good diastereoselectivities (up to 20:1 dr).

**Scheme 12:**
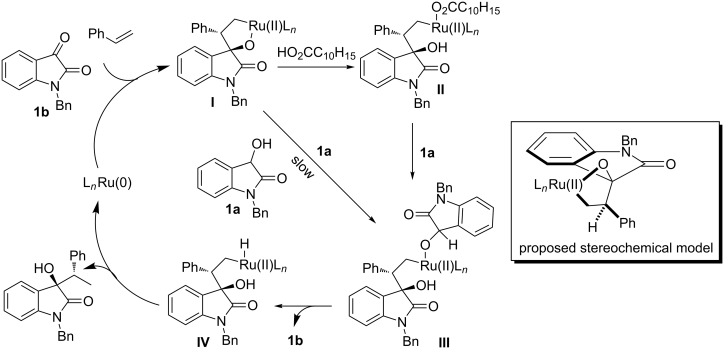
Proposed catalytic mechanism and stereochemical model.

A plausible catalytic mechanism was proposed as shown in Scheme 12. [Ru_3_(CO)_12_] and PCy_3_ formed the ruthenium(0) complex, which then mediated the oxidative coupling of *N*-benzyl-protected isatin and styrene to form oxametallacycle **I**. The ruthenium alkoxide **III** was formed through two possible pathways: (a) the direct transfer hydrogenolytic cleavage of **I** (slow); (b) the protonolysis of **I** by adamantanecarboxylic acid, followed by exchange of the carboxylate **II** with 3-hydroxy-2-oxindole (rapid). β-Hydride elimination of **III** generated ruthenium hydride **IV** and *N*-benzylisatin. Subsequent C–H reductive elimination of **IV** produced the product and regenerated the ruthenium(0) complex. The stereogenetic outcome could be explained by the configuration of oxametallacycle **I**, in which two bulky phenyl rings are *trans* orientated to each other.

**Scheme 13:**
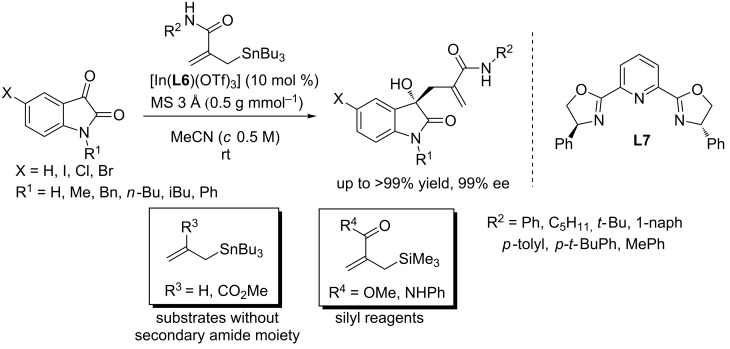
In-catalyzed allylation of isatins with stannylated reagents.

In 2014, Yoda and co-workers developed an indium/PyBox complex-catalyzed enantioselective allylation of isatins with stannylated reagents, affording the products in excellent yields (up to >99%) and with excellent enantioselectivities (up to 99% ee, Scheme 13) [28]. The reactions were performed in acetonitrile at room temperature using 10 mol % of [In(**L7**)(OTf)_3_] as the precatalyst. A wide range of stannylated reagents and isatin derivatives were examined under this system to produce the desired products with excellent results. Interestingly, the secondary amide moiety of the stannylated reagents was proved to be crucial for this reaction. Stannylated reagents without the secondary amide moiety gave unsatisfactory results (≈22% ee). Similarly, silyl reagents also failed to afford the desirable products under the optimized conditions.

**Scheme 14:**
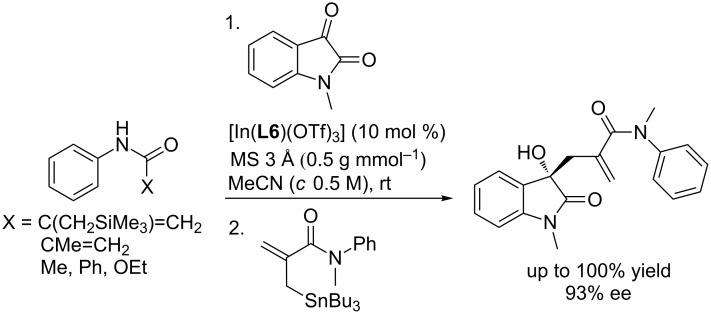
Modified protocol for the synthesis of allylated 3-hydroxyoxindoles.

Based on above observations and understandings toward the mechanism, a modified protocol was designed by introducing the secondary amide containing reagents such as allylsilanes, carbamates, etc, where the amide NH formed a hydrogen bond interaction with isatin. Stannylated reagents attacked isatins from the less steric *Re* face. This modified protocol afforded the desired products in significantly improved yields (up to 100%) and with excellent enantioselectivities (up to 93% ee, Scheme 14).

**Scheme 15:**
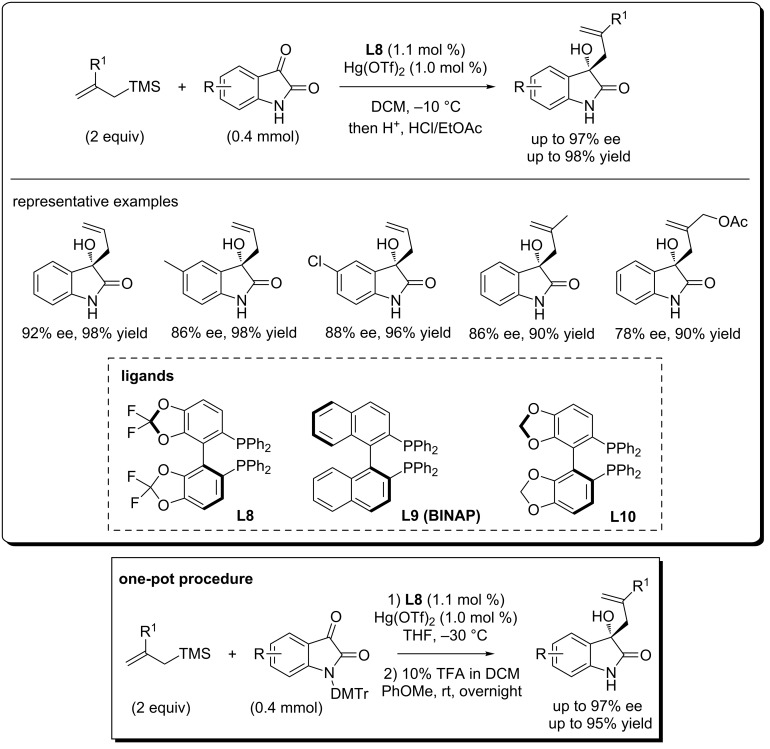
Hg-catalyzed asymmetric allylation of isatins with allyltrimethylsilanes.

Similar to Song’s work [14], Zhou et al. reported that the (*S*)-difluorophos- (**L8**)-derived chiral Hg(OTf)_2_ complex can catalyze the Sakurai–Hosomi reaction of isatins with allyltrimethylsilanes, affording the (*S*)-3-allyl-3-hydroxyoxindoles in up to 92% ee and 98% yield (Scheme 15) [29]. For this reaction, the ligands were found to be important in determining the reactivity and enantioselctivity. When BINAP (**L9**) and the electron-rich ligand **L10** were used, low yields and ee evalues were observed (data not shown here). Additonally, the title compouds can also be obtained in good yields (up to 95% yield) and with excellent ee values (up to 97% ee) from DMTr (Di(*p*-methoxyphenyl)phenylmethyl)-*N*-protected isatins in one-pot under modified conditions.

### Organocatalyzed synthesis of 3-substituted 3-hydroxyoxindoles

Organocatalysis has witnessed significant progress in the last decades, a large number of new organocatalysts have been developed and used in the synthesis of natural products and pharmaceutical agents. Biologically important 3-hydroxyoxindoles with a chiral quaternary center at the 3-position have been successfully accessed utilizing the asymmetric organocatalysis in the last few years. To show recent progress in this field, the asymmetric catalytic synthesis of 3-hydroxyoxindoles are summarized based on the organocatalysts used.

#### Amino acid-derived organocatalysts

**Scheme 16:**
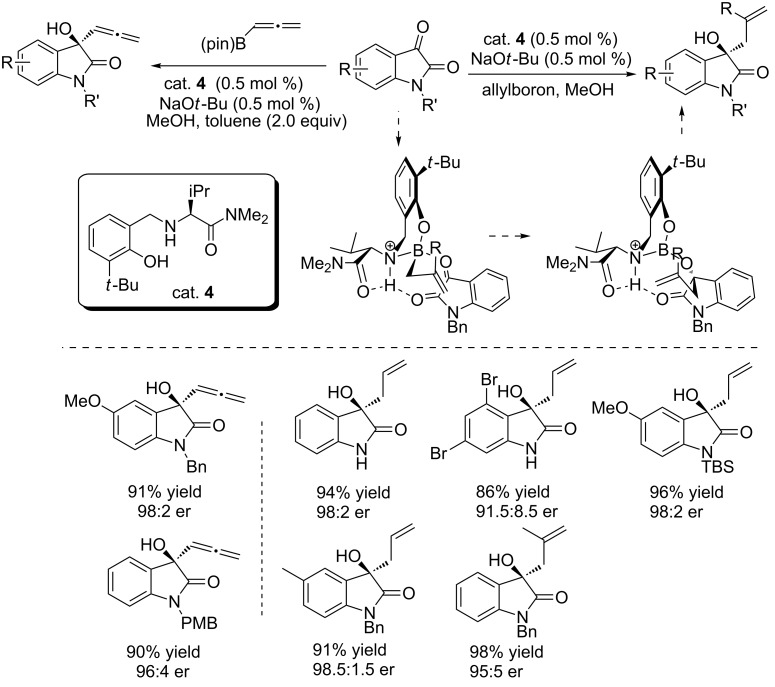
Enantioselective additions of organoborons to isatins.

Amino acids (AAs) have been widely used to develop novel AAs-based organocatalysts for asymmetric synthesis [30]. Representative examples are proline and its derivatives [31]. Recently, Hoveyda and co-workers designed a novel amino acid amide catalyst (cat. **4**) for the asymmetric addition of unsaturated organoboranes to carbonyls and imines, producing the corresponding enantiomerically pure alcohols and amines in excellent yields [32]. As shown in Scheme 16, cat. **4** was successfully applied to the enantioselective addition of allenylboron reagents to isatins, affording homoallylic alcohols in good to moderate yields (up to 98% yield) and with excellent enantioselectivities (up to 98.5:1.5 er). Only 0.5% of catalyst loading and 0.5% NaO*t*-Bu were used for the reaction. The phenolic hydroxy group of the catalyst was thought to be crucial in controlling the enantioselectivity by forming hydrogen bonds with reactants. Besides, enantioselective additions of allenes to isatins were also achieved under these conditions, giving the desired products in good yields and with excellent enantioselectivities (Scheme 16).

**Scheme 17:**
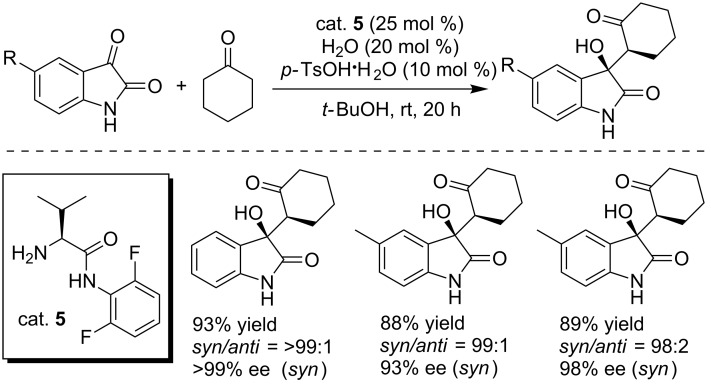
Asymmetric aldol reaction of isatins with cyclohexanone.

In 2013, Ishimaru and co-workers developed a *N*-aryl-L-valinamide (cat. **5**)-catalyzed asymmetric aldol reaction of isatins with ketones, affording the products in excellent yields and with up to >99% ee (Scheme 17) [33]. The reaction was performed under mild conditions using PTSA·H_2_O as the additive.

**Scheme 18:**
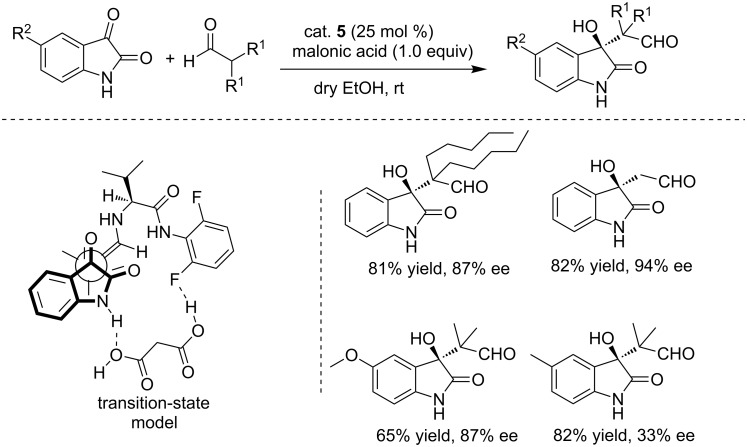
Enantioselective aldol reactions of aliphatic aldehydes with isatin derivatives and the plausible transition-state model.

Subsequently, the asymmetric aldol reaction of aliphatic aldehydes with isatins was achieved by the same group by using a structurally slightly modified organocatalyst (cat. **5**, Scheme 18) [34]. Malonic acid as the additive and anhydrous EtOH as the solvent were found to be critical for achieving high yields and enantioselectivities. However, only 33% ee was obtained for 5-methylisatin under the same conditions. A plausible transition-state model was proposed and examined by DFT calculations, in which malonic acid formed two hydrogen bonds with organocatalyst (cat. **5**) and isatin substrate.

**Scheme 19:**
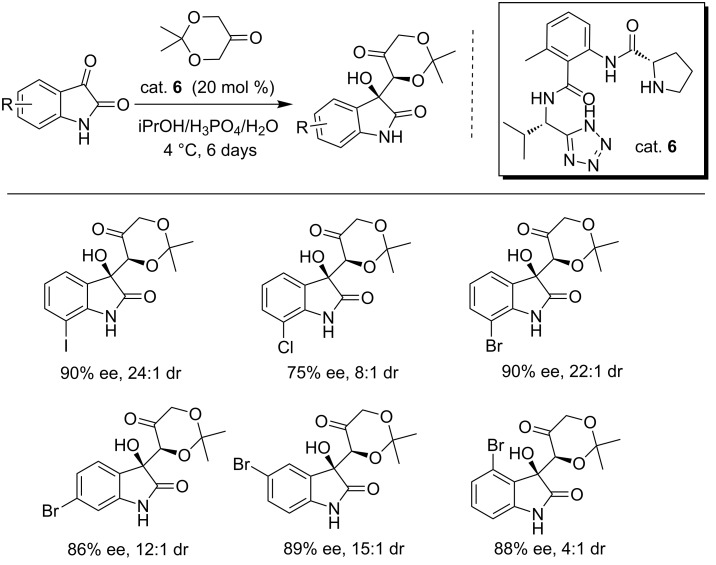
Enantioselective aldol reaction of isatins and 2,2-dimethyl-1,3-dioxan-5-one.

A novel *N*-prolinylanthranilamide-based pseudopeptide organocatalyst (cat. **6**) was designed by Bunge et al. for the enantioselective aldol reaction of 2,2-dimethyl-1,3-dioxan-5-one with isatins, affording the products in good yield and with good enantioselectivities and diastereoselectivies (Scheme 19) [35]. The tetrazole unit and the secondary amide of the catalyst in cat. **6** were the key moieties for the good stereoselectivity. However, the reactions required long reaction times (up to 6 days). This protocol was then successfully applied to the total synthesis of natural TMC-95A.

**Scheme 20:**
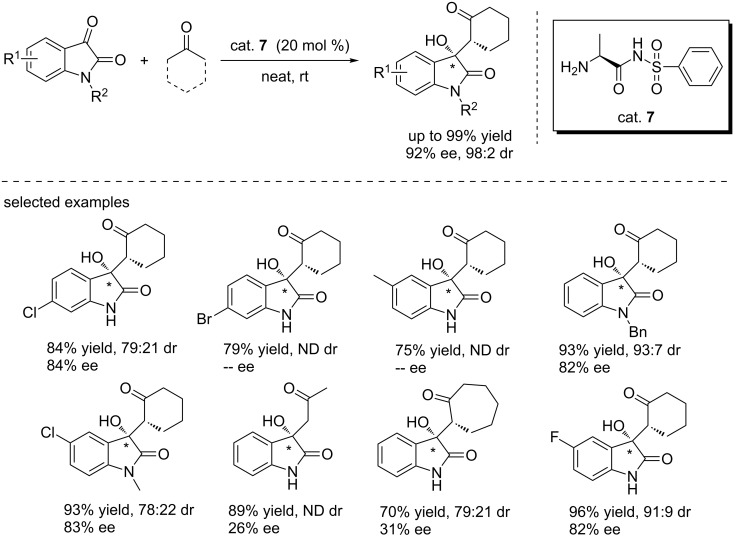
Asymmetric aldol reactions between ketones and isatins.

In 2015, Yang and co-workers reported α-amino acid sulfonamide (cat. **7**)-catalyzed aldol reactions of ketones with isatins under neat conditions (Scheme 20) [36]. Interestingly, the reactions proceeded smoothly, giving the desired products in high yields (up to 99%) and with high enantioselectivities (up to 92% ee) and diastereoselectivities (up to 98:2 dr) using 4 Å molecular sieves as the additive. Various isatin derivatives and ketones (acetone, cyclopentanone and cycloheptanone) were examined, showing that substituents on the isatins had remarkable effects on the enantioselectivity. Compared to acetone and cycloheptanone, good enantioselectivities were obtained for cyclohexanone.

**Scheme 21:**
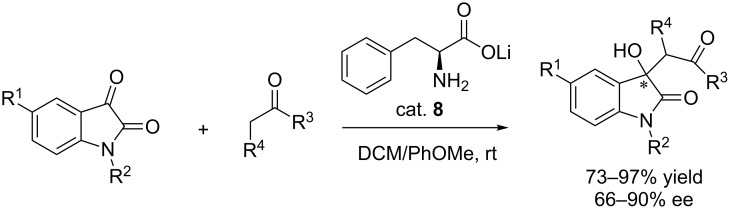
Phenylalanine lithium salt-catalyzed asymmetric synthesis of 3-alkyl-3-hydroxyoxindoles.

Based on the previously observed catalytic performance of amino acid metal salts in asymmetric catalysis, Luo and co-workers reported that phenylalanine lithium salt (cat. **8**) can efficiently catalyze the aldol reaction of substituted isatins with ketones, allowing facile access to the 3-hydroxyoxindoles in moderate to good yields (73–97% yield) and with good enantioselectivity (66–90% ee, Scheme 21) [37]. The phenylalanine lithium salt was easily prepared from phenylalanine and LiOH and the ratios of phenylalanine and LiOH were found to be crucial in determining the enantioselectivity; an excess of LiOH caused racemization of the final products. Besides, the solvent, catalyst loading, the reaction time and temperature were also important for the reaction. Substituents attached to the isatins and ketones had little effect on the yields and enantioselectivity, regardless of their electronic properties and positions.

#### Amino alcohol catalysts

**Scheme 22:**
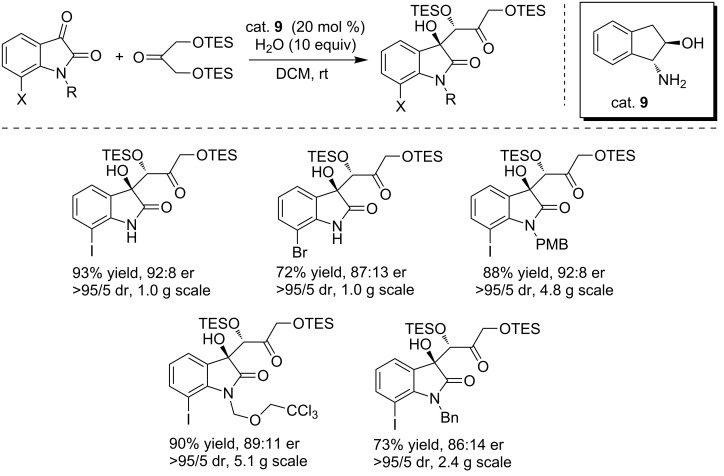
Aldolization between isatins and dihydroxyacetone derivatives.

In 2014, Evano and co-workers reported a concise synthesis of the macrocyclic core of TMC-95A using the asymmetrc aldol addition of isatins with dihydroxyacetone derivatives as the key step (Scheme 22) [38]. The reactions were performed in DCM at room temperature with H_2_O as the co-solvent and cat. **9** as the catalyst, generating the aldol products in good yields and high diastereoselectivities. It is worth mentioning that the aldolization step can be carried out on a multigram scale.

**Scheme 23:**
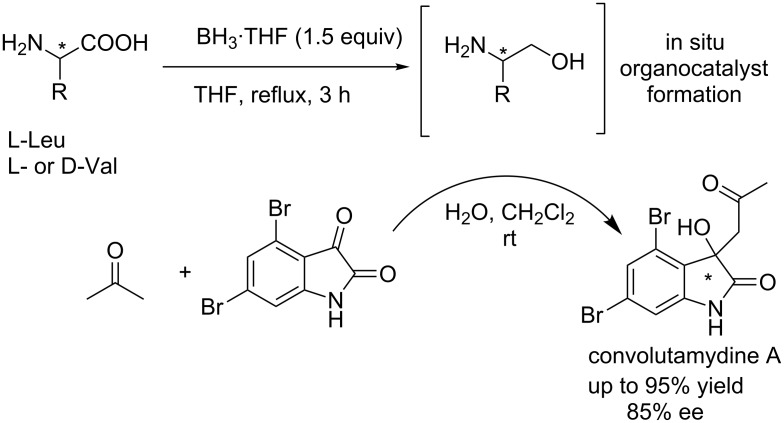
One-pot asymmetric synthesis of convolutamydine A.

Very recently, Tsogoeva and co-workers reported the amino alcohol-catalyzed one-pot enantioselective synthesis of antileukaemia agent (*R*)-convolutamydine A in 95% yield and with 85% ee under mild conditions (Scheme 23) [39]. Notably, the amino alcohol catalysts were generated in situ from the corresponding amino acids L-Leu or L-Val through the BH_3_-mediated reduction, making the multi-step synthesis more efficient and environmentally friendly.

#### Diamine catalysts

**Scheme 24:**
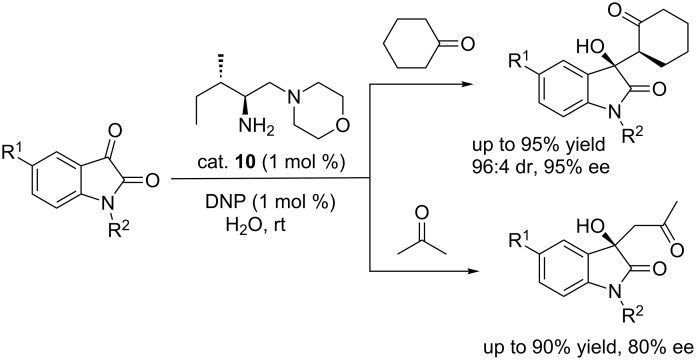
Asymmetric aldol reactions of cyclohexanone and acetone with isatins.

In 2013, Chimni and co-workers reported the diamine catalyst (cat. **10**)-catalyzed aldol reactions of cyclohexanone and acetone with isatins, giving 3-substituted-3-hydroxy-2-oxindoles in excellent yields and with high enantioselectivities (Scheme 24) [40]. It should be noted that the reactions were performed in H_2_O at room temperature using only 1% of catalyst loading. They also found that an increase of catalyst loading led to lower enantioselectivity.

**Scheme 25:**
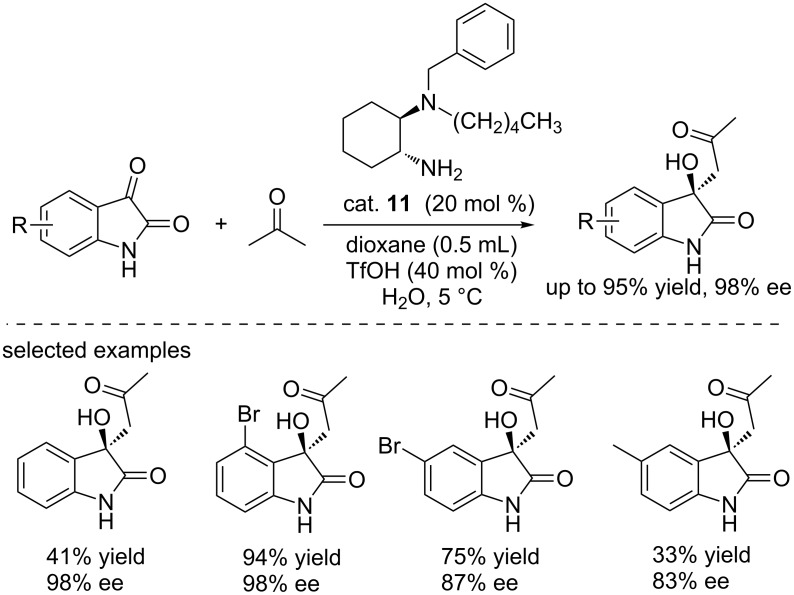
Aldol reactions of acetone with isatins.

Similarly, Gou et al. reported the asymmetric aldol reactions of ketones with isatins catalyzed by a novel 1,2-diaminocyclohexane (DACH)-derived chiral catalyst (cat. **11**, Scheme 25) [41]. The products were obtained in good yields (up to 95%) and with good enantioselectivities (up to 98% ee). Trifluoromethanesulfonic acid and water were proved to be able to improve the yields. This protocol was then successfully applied to the synthesis of (*R*)- and (*S*)-isomers of convolutamydine A.

**Scheme 26:**
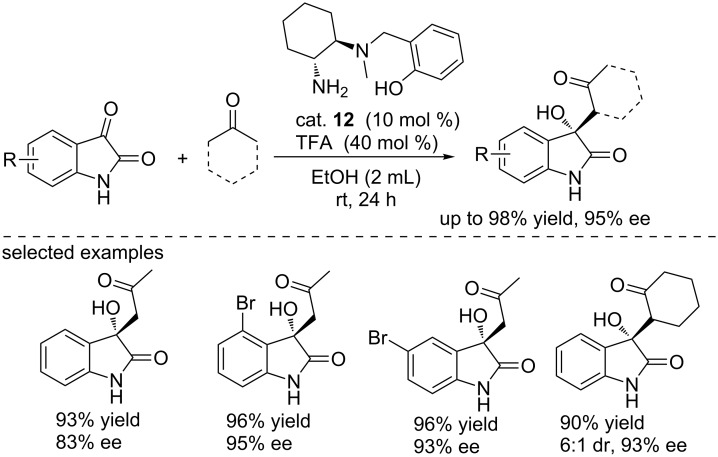
Aldol reactions of ketones with isatins.

Based on the above success, the Gou group designed another DACH-derived organocatalyst cat. **12** bearing an additional phenolic hydroxy group for the asymmetric aldol reactions of isatins with ketones. Cat. **12** can efficiently catalyze the reactions, affording the final compounds in excellent yields (up to 98% yield) and with good enantioselectivities (up to 95% ee, Scheme 26) [42]. The hydroxy group acted as an electron transporter and formed the hydrogen bond with substrates. This hydroxy group played an important role in controlling the enantioselectivity.

#### Squaramide catalysts

**Scheme 27:**
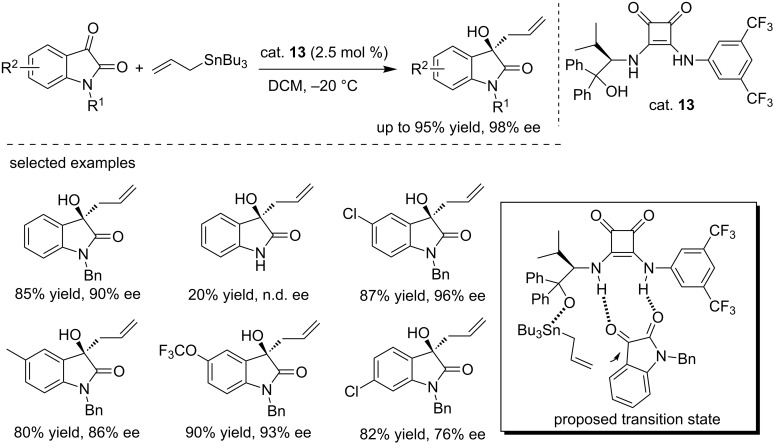
Enantioselective allylation of isatins.

Bajaj and co-workers reported the first squaramide (cat. **13**)-catalyzed enantioselective allylation of isatins with allyltributyltins, producing the desired allylation products in good yields (up to 95%) and with excellent enantioselectivities (up to 98% ee) under mild conditions (Scheme 27) [43]. Only 2.5% of catalyst loading was used in the reactions. However, unprotected isatins failed to gave the desired products under the same conditions. As shown in the binding model, the catalyst activated the carbonyl groups through hydrogen bonds, while the allyltributyltin reagent attacked the 3-carbonyl group from the *Si* face, forming (*R*)-isomers.

#### *Cinchona* alkaloid catalysts

*Cinchona* alkaloids and their derivatives have been widely used as chiral catalysts in many reactions [44] and also show synthetic utilities in the synthesis of enantiomerically pure 3-substituted 3-hydroxyoxindoles.

**Scheme 28:**
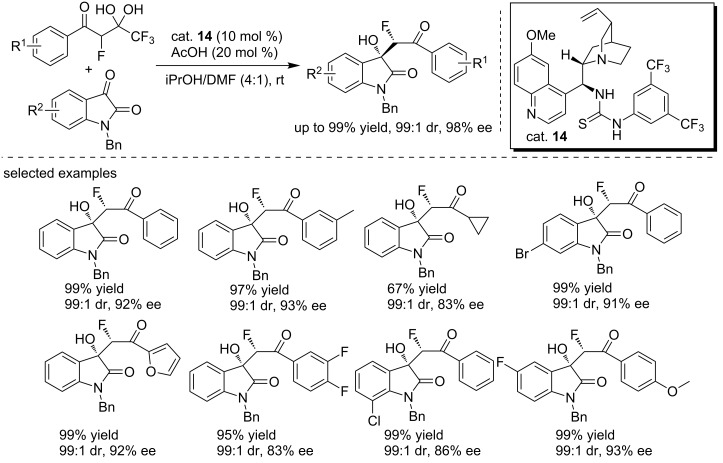
Asymmetric aldol reaction of trifluoromethyl α-fluorinated β-keto gem-diols with isatins.

In 2013, Wu and co-workers reported the *cinchona* alkaloid (cat. **14**)-catalyzed asymmetric aldol reaction of trifluoromethyl α-fluorinated β-keto *gem*-diols with isatins, generating 3-hydroxyoxindoles in high yields (up to 99%) and with excellent enantioselectivities (up to 98% ee, Scheme 28) [45]. The reaction was performed at room temperature using AcOH as the additive in the presence of 10% of catalyst loading. Interestingly, the protocol employed *gem*-diols as equivalents of fluorinated aryl/alkyl methyl ketone enolates for the C–C bond formation accompanied the release of trifluoroacetate. This method showed a broad substrate scope including *gem*-diols with phenyl rings, heterocyclic groups and aliphatic groups.

**Scheme 29:**
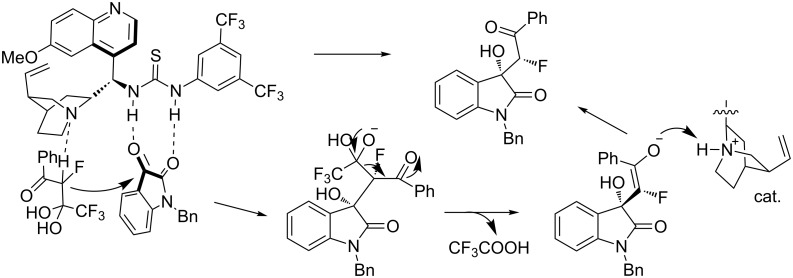
Plausible mechanism proposed for the asymmetric aldol reaction.

A plausible mechanism was proposed as shown in Scheme 29. The thiourea NH moieties and the tertiary amino group of the catalyst activated the carbonyl groups of isatin and the α-protons of *gem*-diols, respectively through hydrogen bond interactions. The α-proton of *gem*-diols was deprived by the amino group of the catalyst, and then the anion attacked isatin from the *Re* face, followed by release of one mol equivalent trifluoroacetate. The enol anion retrieved the proton from the catalyst to give the (*S*)-products.

**Scheme 30:**
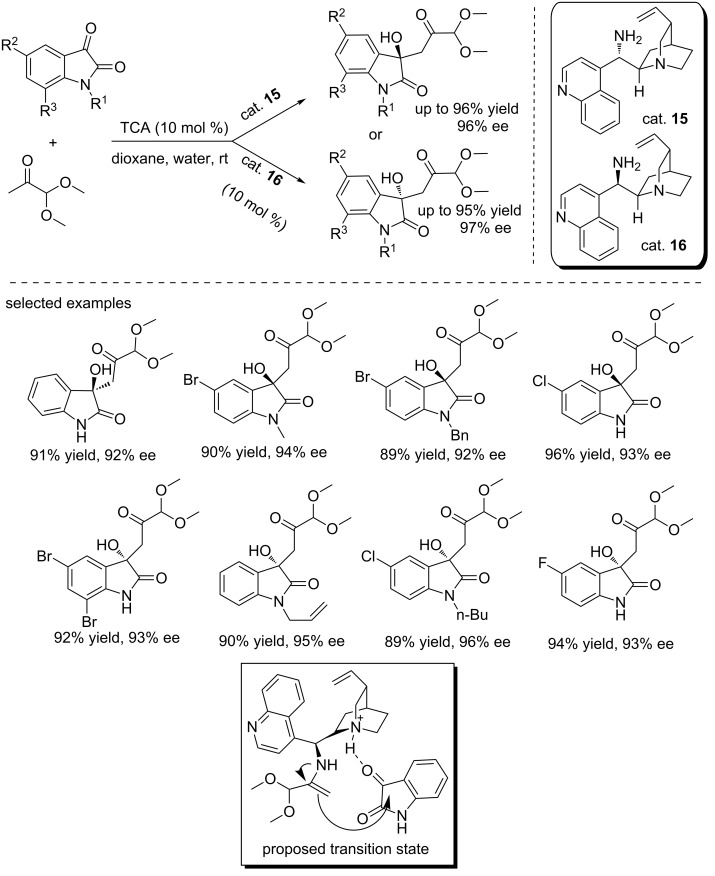
Asymmetric aldol reaction of 1,1-dimethoxyacetone with isatins.

In 2013, Chimni and co-workers designed the *cinchona*-derived primary amine catalysts (cat. **15** and **16**) and achieved the asymmetric aldol reaction of 1,1-dimethoxyacetone with isatins (Scheme 30) [46]. The reactions were carried out at room temperature using 10% catalyst loading and trichloroacetic acid (TCA) as the additive, affording the corresponding 3-hydroxyoxindoles in excellent yields (up to 96% yield) and with high enantioselectivies (up to 97% ee), even for *N*-unsubstituted isatin derivatives. Besides, this protocol showed a broad substrate scope and good tolerance toward different functional groups. TCA promoted the formation of an enamine from the primary amine of the catalyst and ketone substrate and protonation of the tertiary amino group. The protonated amine then served as hydrogen bond donor to activate the carbonyl group of isatin substrates, thereby facilitating the aldol addition. Interestingly, the authors obtained the *R-/S*-enantiomer by using the corresponding *R-/S*-organocatalyst, respectively. The stereoselectivity could be explained by the transition state proposed. The *R*-enamine formed from the corresponding *R*-catalyst and 1,1-dimethoxyacetone attacked the isatin substrate from the *Re* face, thus affording the *R*-enantiomer.

**Scheme 31:**
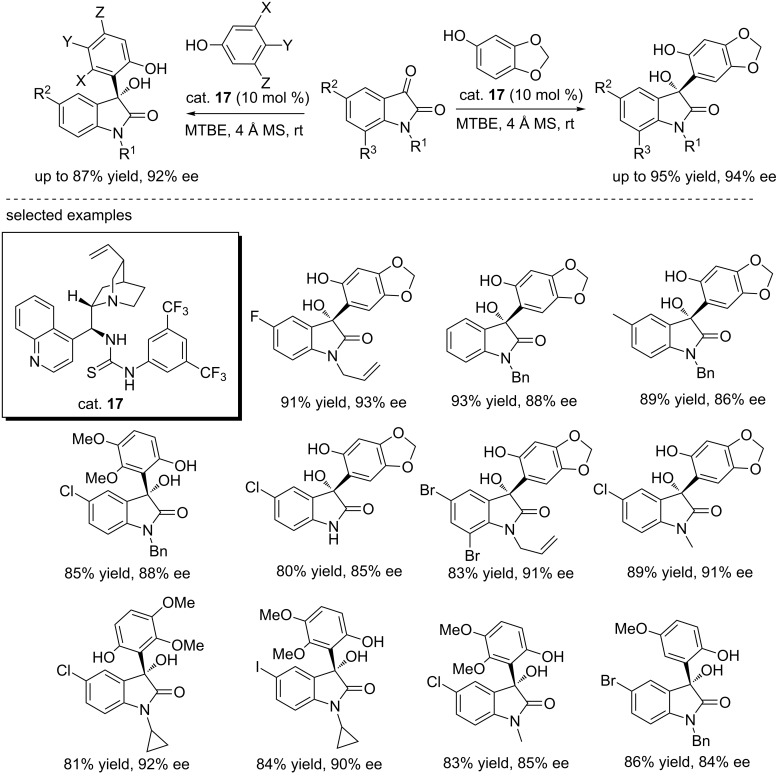
Enantioselective Friedel-Crafts reaction of phenols with isatins.

**Scheme 32:**
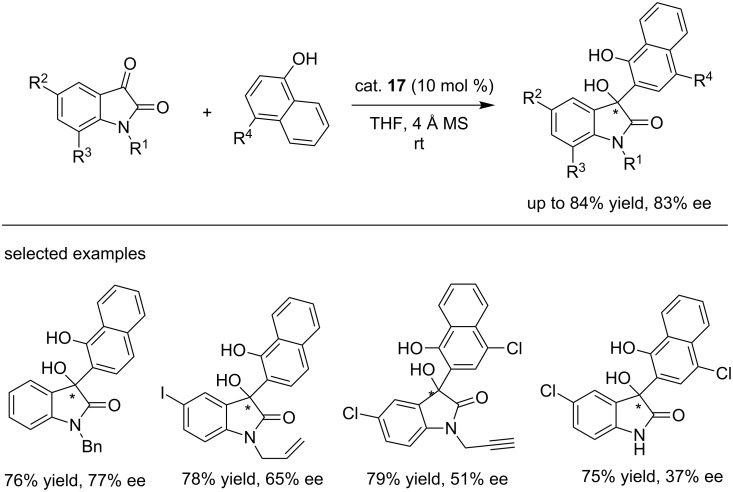
Enantioselective addition of 1-naphthols with isatins.

In 2014, the same group reported the organocatalytic asymmetric addition reactions of isatins with electron-rich aromatics sesamols using the *cinchona* alkaloid-derived thiourea catalyst (cat. **17**) under mild conditions. In the presence of 10% catalyst, sesamols reacted with isatins smoothly in *tert*-butyl ether at room temperature, generating a variety of 3-aryl-3-hydroxy-2-oxindoles in good yields (up to 95%) and with good enantioselectivities (up to 94% ee), even for *N*-unsubstituted isatins (Scheme 31) [47]. These conditions were also successfully extended to other phenol substrates, affording the corresponding (*R*)-3-aryl-3-hydroxyoxindoles in good yields (up to 87% yield) and with excellent enantioselectivity (up to 92% ee, Scheme 31) [48]. This slightly modified protocol was then successfully applied to the addition reactions of 1-naphthols and isatins, affording the desired products in slightly lower yields (70–84%) and with decreased enantioselectivities (37–83% ee, Scheme 32) [49].

**Scheme 33:**
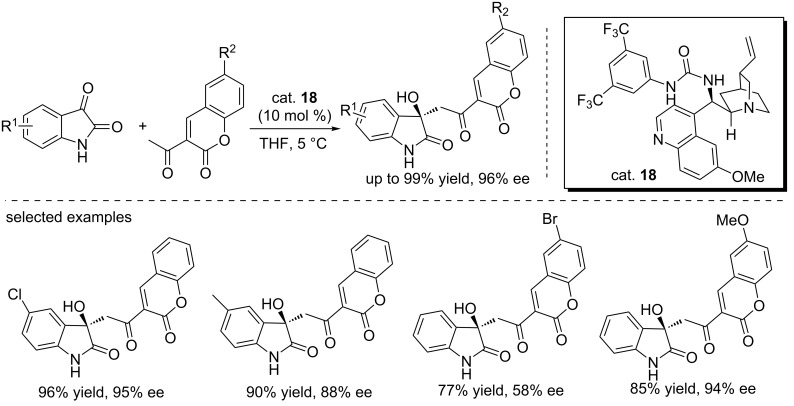
Enantioselective aldol reaction between 3-acetyl-2*H*-chromen-2-ones and isatins.

Zhao and co-workers developed the first enantioselective aldol reaction of 3-acetyl-2*H*-chromen-2-ones with isatins catalyzed by the quinidine-derived urea catalyst (cat. **18**, Scheme 33) [50]. The reactions were performed in THF at low temperature (5 °C), affording final compounds in moderate to excellent yields (up to 99%) and with moderate to high enantioselectivities (up to 96% ee). It was proved that substituents on the isatin derivatives had little influence on the reaction rate and enantioselectivity, while 3-acetyl-2*H*-chromen-2-ones bearing an electron-withdrawing group gave the products in lower yields and with low ee values. In contrast, good reactivity and high enantioselectivity were observed for those with a electron-donating methoxy group probably due to the electronic effects. The final products contain two biologically important structural scaffolds, namely the 3-hydroxyoxindole and coumarin, which could be potentially used for further biological evaluation or serve as starting points in drug discovery pursuits.

**Scheme 34:**
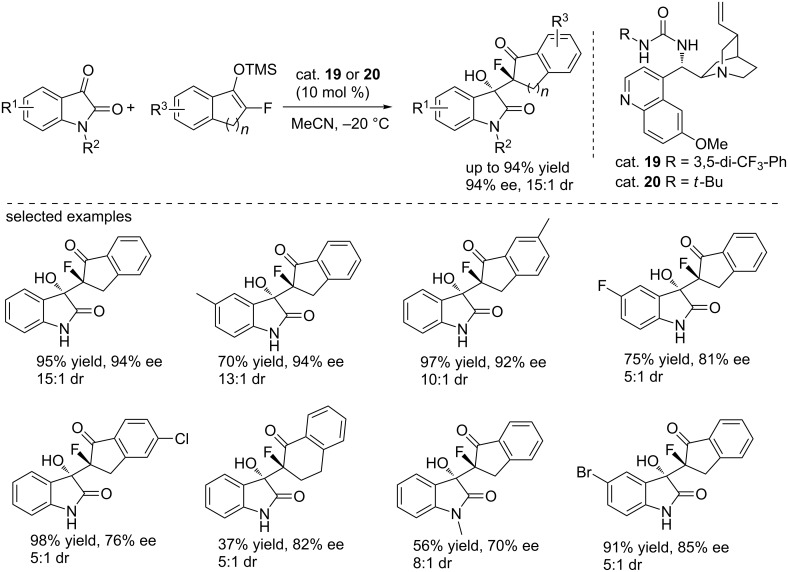
Stereoselective Mukaiyama–aldol reaction of fluorinated silyl enol ethers with isatins.

A highly stereoselective Mukaiyama–aldol reaction was reported by Zhou and co-workers in 2014 (Scheme 34) [51]. After activation by the tertiary amine catalysts (cat. **19** and **20**), the monofluorinated silyl enol ether then reacted with isatins to generate 3-hydroxyoxindoles bearing two adjacent chiral carbon centers. The reactions were performed in acetonitrile with 10 mol % of catalyst loading at –20 °C. They also highlighted another *cinchona* alkaloid-derived urea catalyst bearing an *N*-alkyl group (cat. **20**), which was more efficient than cat. **19** in the reactions with halogenated isatins. Compared to *N*-unsubstituted isatins, the *N*-methyl-protected isatin gave a lower yield and ee value under the same conditions, suggesting that the NH moiety is critical for achieving good results. Under the same conditions, the α-fluorotetralone-derived silyl enol ether gave the corresponding product in 37% yield and with 82% ee.

**Scheme 35:**
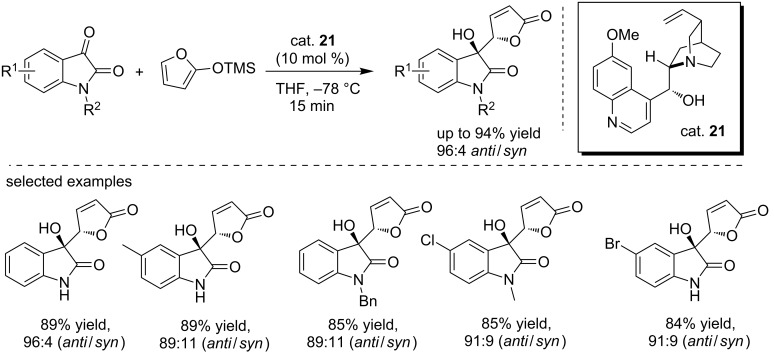
Asymmetric vinylogous Mukaiyama–aldol reaction between 2-(trimethylsilyloxy)furan and isatins.

More recently, Miao and co-workers reported the quinidine (cat. **21**)-catalyzed vinylogous Mukaiyama–aldol reaction between 2-(trimethylsilyloxy)furan and isatins (Scheme 35) [52]. The reactions were performed in THF at −78 °C, affording the corresponding addition products in high yields (up to 94%) and with good diastereoselectivities (the ratio of *anit*/*syn* is up to 96:4). The generality of this protocol was examined using various isatin derivatives and all reactions proceeded smoothly within 15 min to give the desired products in good results regardless of the electronic properties of the substituents. Additionally, the *N*-protecting group of isatins was found to have a certain influence on the reactivity. Interestingly, the final compounds bear the 3-hydroxyoxindole and butenolide moieties and could be used for biological screening or serve as intermediates for further transformations.

**Scheme 36:**
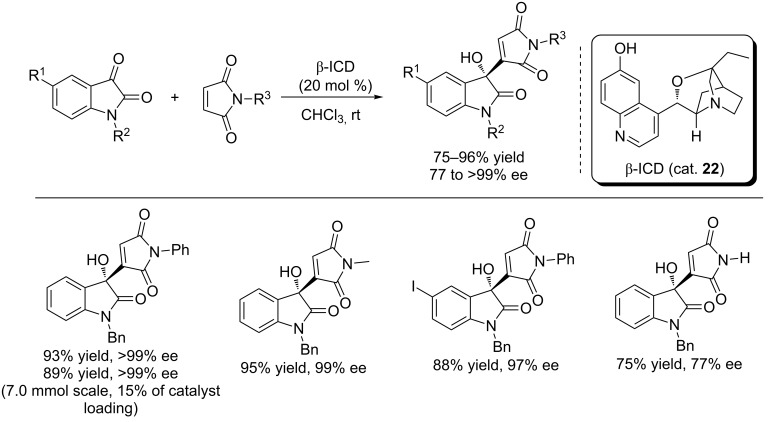
β-ICD-catalyzed MBH reactions of isatins with maleimides.

After screening different chicona alkanoid-derived organocatalysts, Chimni et al. found that β-isocupreidine (β-ICD, cat. **22**) can efficiently catalyze the Morita–Baylis–Hillman (MBH) reaction of isatins with maleimides to generate the 3-substituted 3-hydroxyoxindoles in excellent yields (up to 96% yield) and with excellent enantioselectivity (up to >99% ee) under mild conditions (Scheme 36) [53]. CHCl_3_ was proved to be a superior solvent for the MBH reactions. Most of the products were obtained in excellent yields and with excelent enantioselectivity, for *N*-unprotected maleimide, the corresponding product was obtained in a lower yield and enantioselectivity (75% yield and 77% ee). Interestingly, a satisfactory result (89% yield and >99% ee) was also obtained when the reaction was performed on a 7.0 mmol scale using 15% of catalyst loading.

**Scheme 37:**
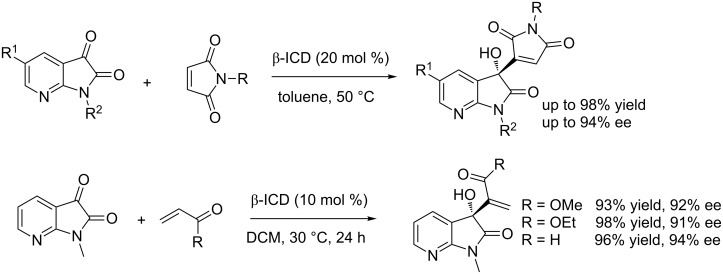
β-ICD-catalyzed MBH reactions of 7-azaisatins with maleimides and activated alkenes.

Similarly, Chen and co-workers reported that β-ICD, as a bifunctional catalyst, can also efficiently catalyze the MBH reactions of 7-azaisatins with maleimides, affording the corresponding products in excellent yields (up to 98% yield) and with excellent enantioselectivity (up to 94% ee, Scheme 37) [54]. Additionally, other activated alkenes such as methyl and ethyl acrylates and acrolein reacted smoothly with 7-azaisatins, giving the corresponding products in excellent yields and with excellent enantioselectivity. The authors found that 7-azaisatins are better electrophiles than isatins and could be used for accessing biologically important isatin analogs.

**Scheme 38:**
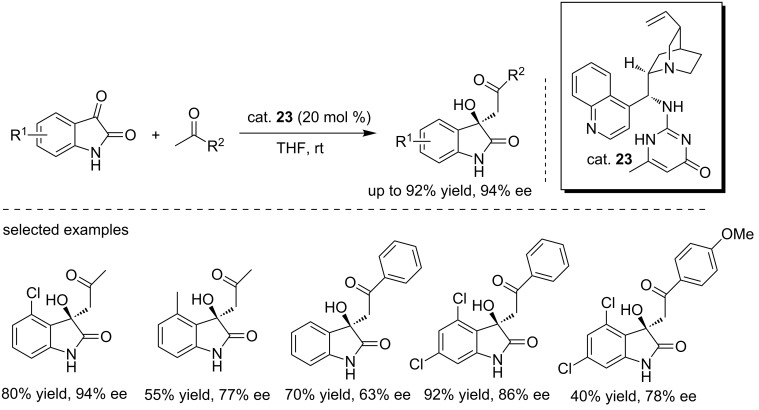
Enantioselective aldol reaction of isatins with ketones.

In 2015, Ren et al. developed a novel quinidine catalyst (cat. **23**) bearing a 2-aminopyrimidin-4(1*H*)-one moiety for enantioselective aldol reactions of isatins with ketones (Scheme 38) [55]. The reactions were conducted in THF with 20 mol % of catalyst loading at room temperature, affording the final compounds in moderate to good yields (up to 92% yield) and with moderate to good enantioselectivities (up to 94% ee). A variety of substituted isatins were well tolerated under these conditions. In addition, the weak nucleophile acetophenone also reacted with isatins, giving the corresponding products with satisfactory results under the same conditions, although acetophenone was always considered to be difficult to isomerize to the enol form. The introduction of halogen substituents at the 4- and 7-positions of isatin was beneficial for the reaction. Acetophenones with electron-withdrawing groups demonstrated high reactivity, while those with electron-donating groups resulted in much lower yields and enantioselectivities.

#### (Thio)urea catalysts

**Scheme 39:**
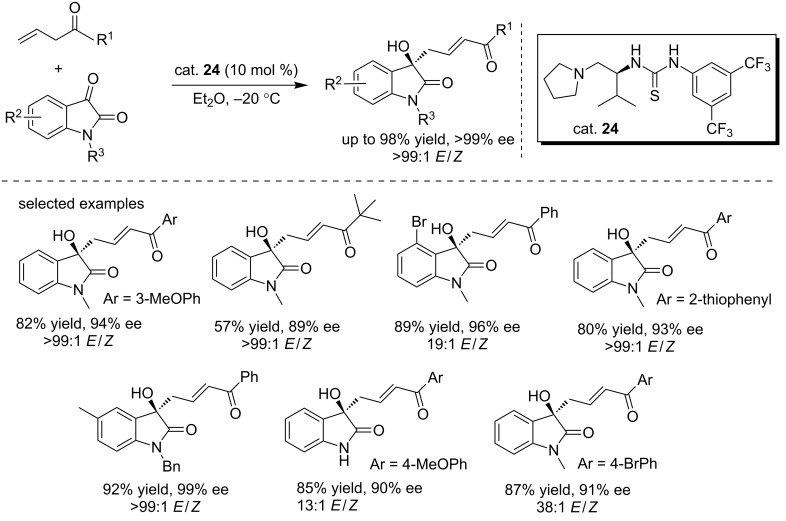
Direct asymmetric vinylogous aldol reactions of allyl ketones with isatins.

Different from the above mentioned aldol reactions, Jiang and co-workers reported the first highly enantioselective vinylogous aldol reaction of allyl ketones with isatins (Scheme 39) [56]. The reactions were performed in diethyl ether in the presence of the bifunctional thiourea catalyst (cat. **24**, 10 mol %), affording the *E*-adducts in high yields (up to 95%) and with moderate to good enantioselectivities (up to 99% ee). Different allyl ketones were examined under these conditions, affording the corresponding products in good yields, especially for the aldol product derived from allyl *tert*-butyl ketone. In addition, *N*-unprotected isatins were also well tolerated. The desired *E-*adducts can be obtained by simple filtration with high ee values (>98% ee). The final compounds could be used as precursors for the synthesis of biologically important spirooxindoles utilizing the hydroxy group and the unsaturated ketone moiety.

**Scheme 40:**
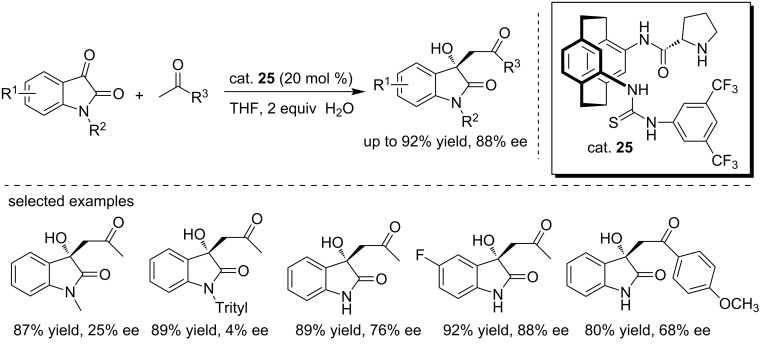
Enantioselective aldol reactions of ketones with isatins.

Song and co-workers designed a novel [2.2]paracyclophane-based thiourea catalyst (cat. **25**), which was successfully applied to the enantioselective aldol reaction of isatins with enolizable ketones using H_2_O as the additive. The desired adducts were obtained in high yields (up to 92%) and with moderate to good enantioselectivities (up to 88% ee, Scheme 40) [57]. The substrate scope was examined, showing that the protecting group attached to the isatin amide was crucial for the enantioselectivity, while the electronic properties of substituents on the isatin aromatic ring had little effect on the reaction. Particularly, when the *N*-protecting group was methyl and triphenylmethyl (trityl), the corresponding products were obtained with extremely low enantioselectivities (25% and 4% ee, respectively).

**Scheme 41:**
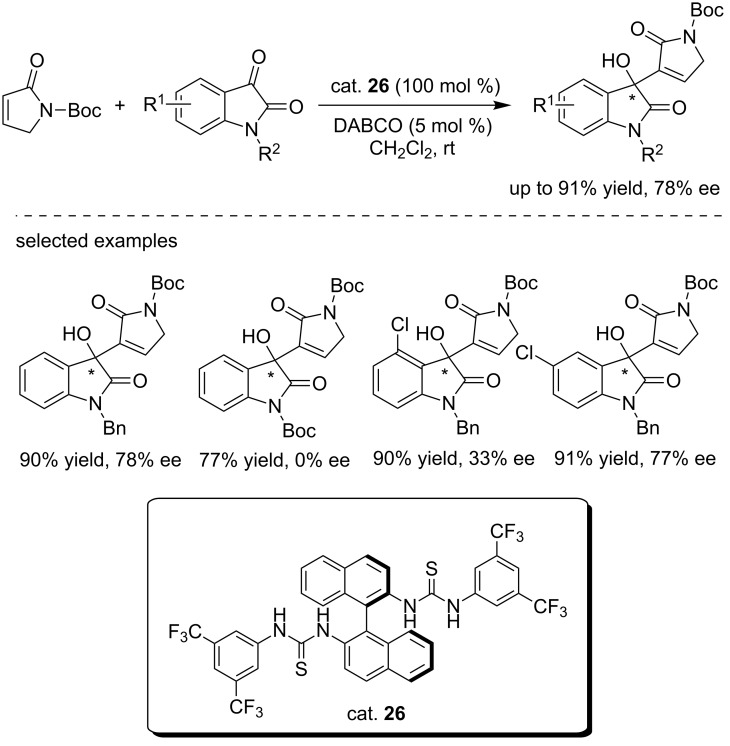
The MBH reaction of isatins with α,β-unsaturated γ-butyrolactam.

Pan and co-workers reported the first bisthiourea (cat. **26**)-catalyzed asymmetric Morita–Baylis–Hillman reaction of isatins with α*,*β-unsaturated γ-butyrolactam (Scheme 41) [58]. The reactions were performed in DCM at room temperature with catalytic amounts of DABCO (5 mol %). A variety of isatin derivatives were tested under this catalytic system, affording the desired products with satisfactory results (up to 91% yield and 78% ee). The *N*-protecting group and substituents on the phenyl ring of isatins were found to be crucial for the reactivity. For the Boc-protected isatin substrate, only the racemic product was obtained possibly because of the unwanted hydrogen bond interactions of the Boc group with the catalyst. The position of the substituent on the phenyl ring of isatin had a remarkable effect on the stereoselectivity. The ee value increased from 33% (4-chloro substituted) to 77% (5-chloro substituted).

**Scheme 42:**
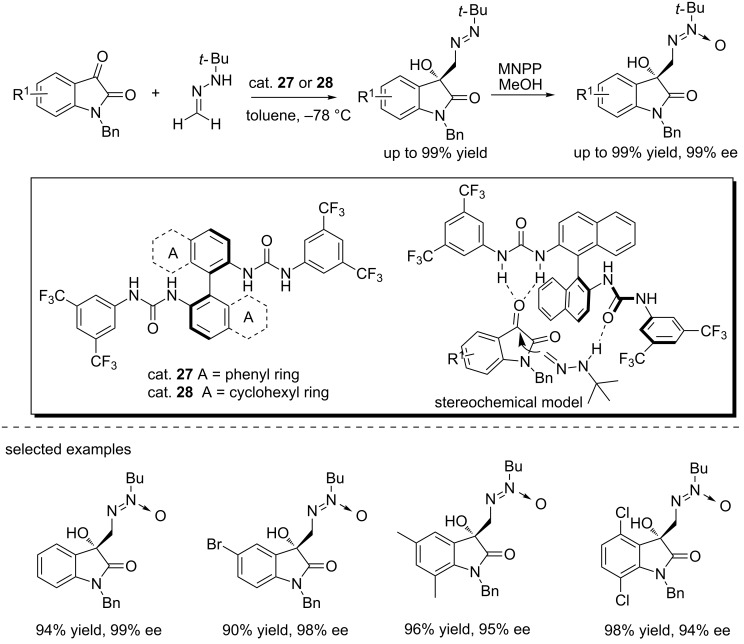
Reactions of *tert*-butyl hydrazones with isatins followed by oxidation.

Similarly, Lassaletta and co-workers designed the bifunctional bisurea catalysts (cat. **27** and **28**) that promoted the reactions of isatins with *tert*-butylhydrazones through hydrogen bond interactions, affording the functionalized 3-hydroxy-2-oxindoles in low enantioselectivity because of the racemization of the adduct products, albeit with high yields (up to 99% yield, Scheme 42) [59]. Interestingly, treatment of the adducts with magnesium monoperoxyphthalate (MMPP) gave the corresponding azoxy compounds in excellent yields (up to 99%) and with complete regioselectivities and excellent enantioselectivities (up to 99% ee). A stereochemical model was proposed to explain the high enantioselectivity, highlighting the pivotal role of the dual activation of the catalyst.

**Scheme 43:**
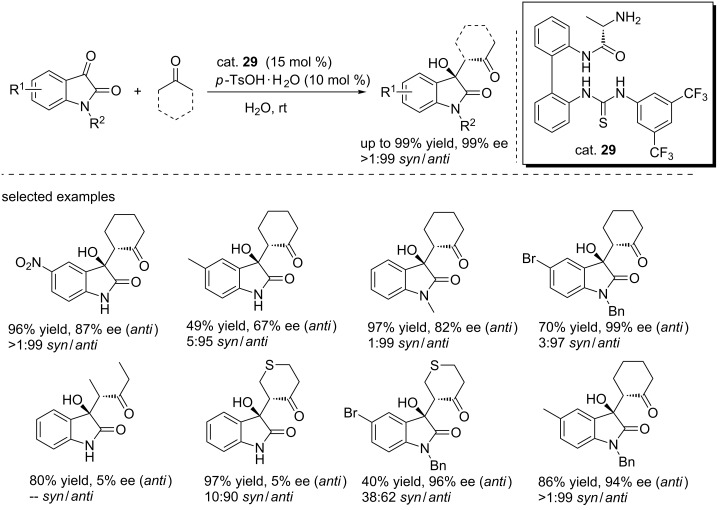
Aldol reactions of isatin derivatives with ketones.

In 2014, Zhao and co-workers designed a series of (thio)urea catalysts (cat. **29**) bearing an axially unfixed biphenyl moiety for the aldol reactions between isatins and enolizable ketones (Scheme 43) [60]. The reactions were performed in H_2_O at room temperature with PTSA·H_2_O (10 mol %) as the additive to deliver the corresponding adducts in high yields (up to 99%) and with good to excellent diastereoselectivities (1:99 dr) and enantioselectivities (up to 99% ee). A broad range of isatin derivatives were tolerated under these conditions. The aldol reaction of *N*-benzyl-5-bromoisatin with 1-thiacyclohexan-4-one and cyclohexanone gave the corresponding products with 96% and 99% ee, respectively.

**Scheme 44:**
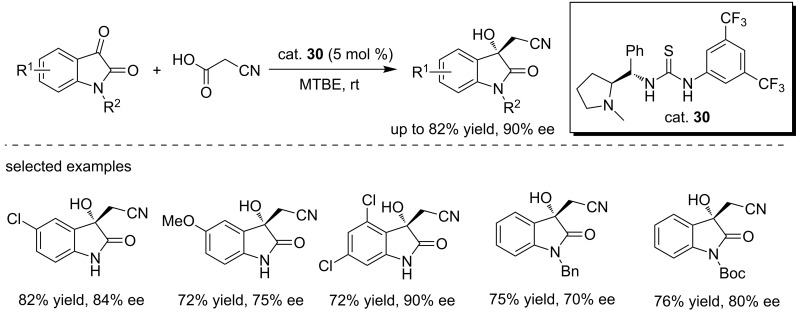
Enantioselective decarboxylative cyanomethylation of isatins.

More recently, Kesavan and co-workers reported an asymmetric decarboxylative cyanomethylation of isatins with cyanoacetic acid using the L-proline-derived thiourea as the catalyst (cat. **30**), giving the cyanomethylated products in good yields (up to 82%) and with excellent enantioselectivities (up to 90% ee, Scheme 44) [61]. The reactions were performed in methyl *tert*-butyl ether at room temperature with 5 mol % of catalyst loading. A variety of isatin derivatives were able to react with cyanoacetic acid to form the products regardless of the electronic properties of substituents on substrates. From our own perspectives, the adjacent OH and cyano groups could be potentially used for constructing biologically important spirooxindoles.

**Scheme 45:**
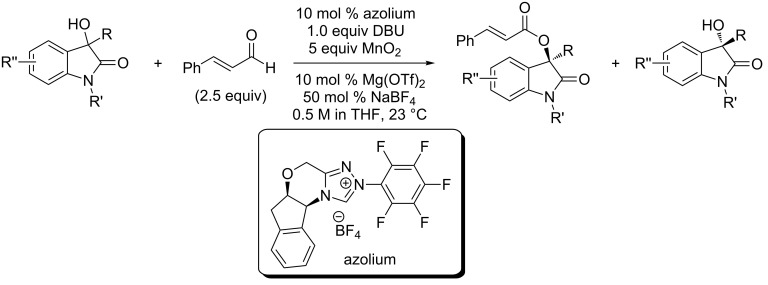
Catalytic kinetic resolution of 3-hydroxy-3-substituted oxindoles.

In addition to the above mentioned metal- and organocatalyzed asymmetric synthesis of 3-hydroxyoxindoles, Zhao et al. reported a complementary strategy that employed the chiral acylazolium species (prepared from aldehydes and *N*-heterocycle carbenes) to achieve the catalytic kinetic resolution of readily available racemic 3-hydroxy-3-substituted oxindoles, giving the enantiopure 3-hydroxyoxindoles with excellent enantioselectivities (Scheme 45) [62]. In this reaction, the oxidant MnO_2_ was considered to be important for the reactvity and selectivity and superior to quinone. The cinnamaldehyde was proved to be more efficient and selective than other aldehydes for the reaction.

#### 3-Hydroxyoxindole-based further transformations

**Scheme 46:**
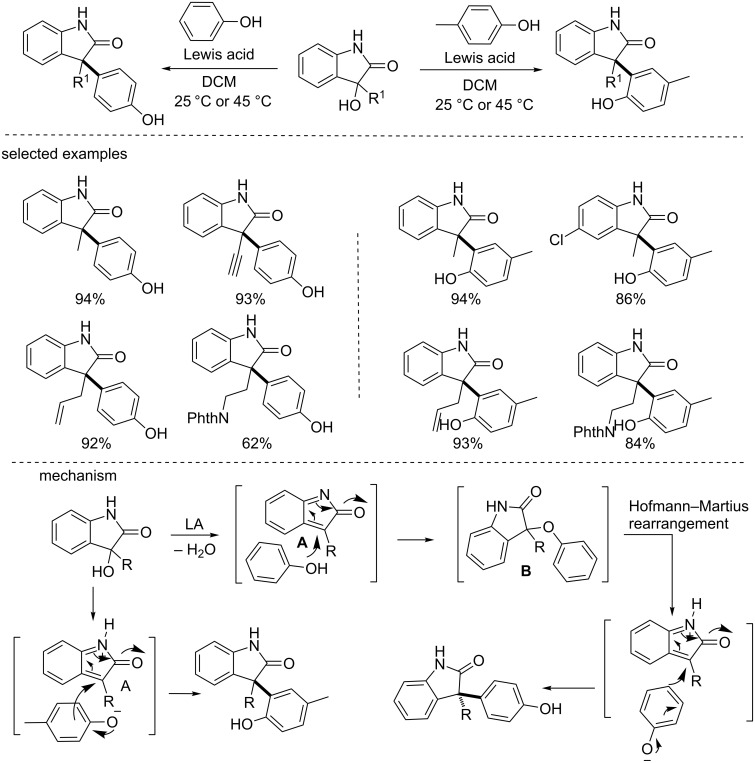
Lewis acid catalyzed Friedel–Crafts alkylation of 3-hydroxy-2-oxindoles with electron-rich phenols.

Apart from aforementioned asymmetric construction of biologically important 3-hydroxyoxindoles, their use as versatile precursors has also been studied for the total synthesis of natural products and accessing biologically important scaffolds. In 2012, Bisai et al. reported the Lewis acid-catalyzed Friedel–Crafts alkylation of 3-hydroxy-2-oxindoles with electron-rich phenols (Scheme 46) [63]. They found that various Lewis acids (e.g., Sc(OTf)_3_, In(OTf)_3_, Zn(OTf)_2_, FeCl_3_, etc) can catalyze the reactions to give 3,3-disubstituted oxindoles in moderate to excellent yields and with excellent regioselectivities. For *para*-substituted phenols, the reactions gave the corresponding *ortho*-substituted products. While for *para*-unsubstituted phenols, the reactions exclusively gave the *para*-substituted products. Mechanistically, in the presence of a catalytic amount of Lewis acid, the 3-hydroxyoxindole was converted to the intermediate **A** through dehydration, which was then tapped by the electron-rich phenol. *para*-Substituted products were formed possibly through two pathways: (a) the direct addition of phenols at the electron-rich *para*-position; (b) the phenolic hydroxy group attacked the intermediate **A** to form the 3-aryloxy-2-oxindole **B**, followed by a Hofmann–Martius rearrangement, affording the *para*-substituted products.

**Scheme 47:**
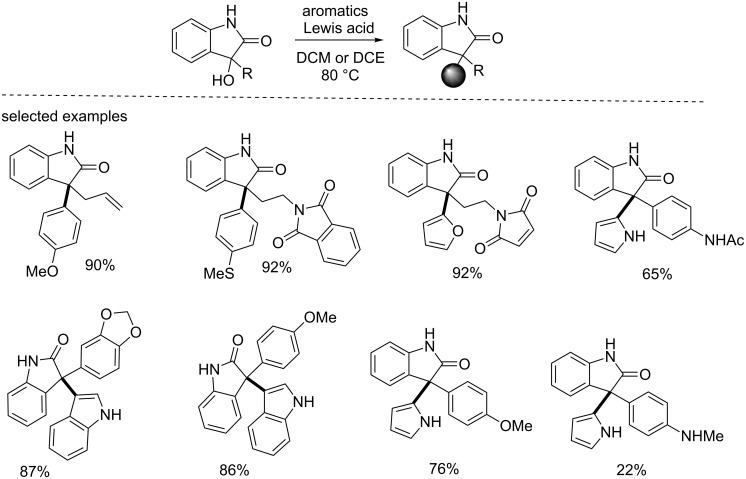
Lewis acid catalyzed arylation of 3-hydroxyoxindoles with aromatics.

Subsequently, the same group extended this protocol to other electron-rich aromatics such as anisole, furan, indole and aniline, generating the corresponding products in good to excellent yields (Scheme 47) [64]. This method may provide a fast entry to the 3-indolyloxindoles (also known as indol-3-ylmethanols), which have been widely used as versatile intermediates for synthesizing biologically important indole containing compounds.

**Scheme 48:**
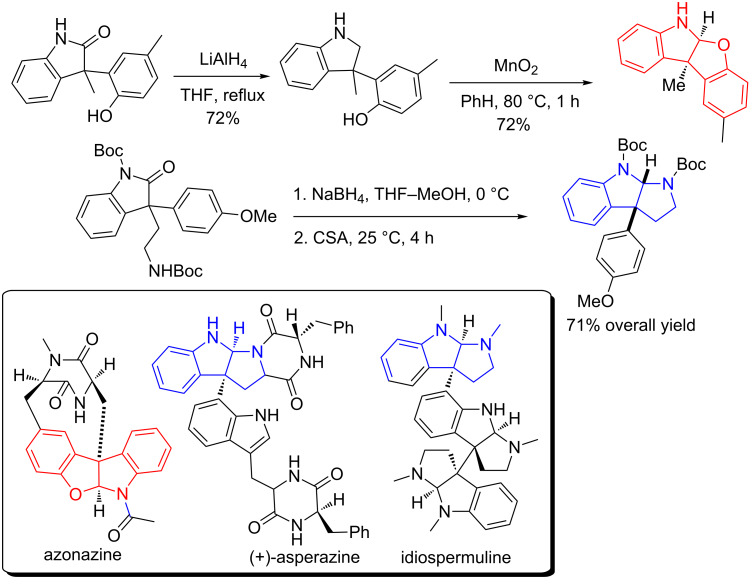
Synthetic application of 3-arylated disubstituted oxindoles in the construction of core structures in natural products.

The method was also successfully applied to the construction of the tetracyclic core that exists in natural azonazine through a reductive cyclization/MnO_2_-mediated intramolecular oxidative coupling sequence (Scheme 48). Similarly, the tricyclic *N*-heterocyclic core in natural (+)-asperazine and idiospermuline was also efficiently synthesized in 71% overall yield. The oxindole was reduced to the lactol by NaBH_4_, which was then subjected to a camphorsulfonic acid (CSA)-promoted cyclization, giving the tricyclic *N*-heterocyclic core.

**Scheme 49:**
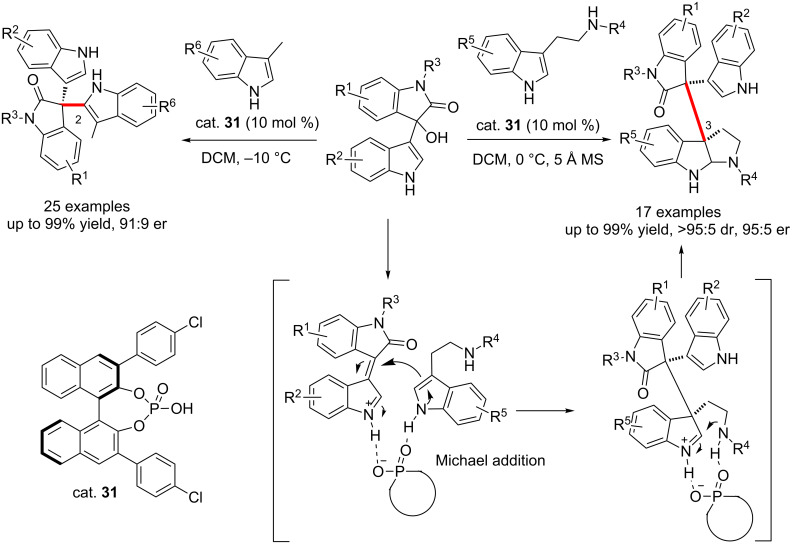
CPA-catalyzed dearomatization and arylation of 3-indolyl-3-hydroxyoxindoles with tryptamines and 3-methyloxindoles, respectively.

Very recently, Shi and co-workers reported the chiral phosphoric acid (CPA, cat. **31**)-catalyzed asymmetric dearomatization reactions of tryptamines with 3-indolyl-3-hydroxyoxindoles, affording the indole-containing tricyclic *N*-heterocycles in a highly stereoselective manner (up to 99% yield, >95:5 dr and 95:5 er, Scheme 49) [65]. The electronic properties of the substituents had a remarkable effect on the yields and enantioselectivities. Mechanistically, the CPA dually activated both substrates through hydrogen-bond interactions. Tryptamines acted as nucleophiles attacking the vinyliminium intermediates generated in situ from the indol-3-ylmethanols (the Michael addition) to form the intermediates. This is followed by the CPA-promoted cyclization, affording the final products bearing three continuous chiral centers. Besides, they also reported the CPA-catalyzed enantioselective arylation of isatin-derived 3-indolylmethanols with 3-methylindoles, affording the biologically important 3,3’-bis(indolyl)oxindoles in excellent yields (up to 99%) and with good enantioselectivities (up to 91:9 er) [66]. The reactions proceeded smoothly in different solvents (DCM, toluene, MeCN, DCE, 1,4-dioxane, EtOAc, etc.) with H_2_O as the waste. A wide range of functional groups were well tolerated. Control experiments showed that the NH group in the 3-indolylmethanols played an important role in controlling the enantioselectivity, while the NH group of 3-methylindoles was crucial for the reactivity by forming hydrogen-bond interactions with CPA.

**Scheme 50:**
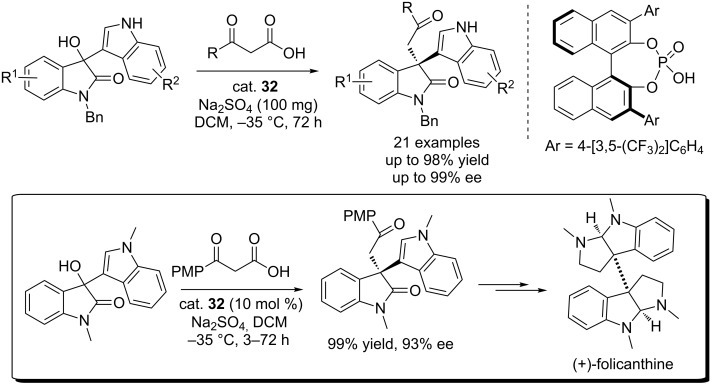
CPA-catalyzed enantioselective decarboxylative alkylation of β-keto acids with 3-hydroxy-3-indolyloxindoles.

Ma and co-workers described the CPA (cat. **32**)-catalyzed enantioselective decarboxylative alkylation of β-keto acids with 3-hydroxy-3-indolyloxindoles, affording the 3-functionlized 3-indolyloxindoles bearing an all-carbon quaternary stereocenter in good yields (up to 98% yield) and with excellent enantioselectivities (up to 99% ee, Scheme 50) [67]. The reactions showed good tolerance to various aromatic and aliphatic β-keto acids as well as substituted 3-hydroxy-3-indolyloxindoles. It was proved that the aryl group at the 3,3’-position of the BINOL backbone had a remarkable effect on the catalytic activity and enantioselectivity. Based on this method, the authors also achieved the synthesis of a key intermediate efficiently (99% yield and 93% ee), which had been used in the total synthesis of (+)-folicanthine [68].

**Scheme 51:**
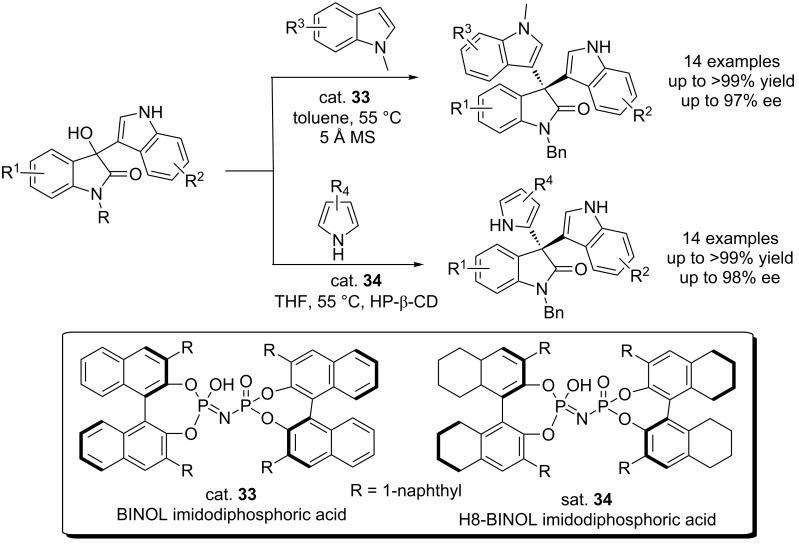
BINOL-derived imidodiphosphoric acid-catalyzed enantioselective Friedel–Crafts reactions of indoles and pyrroles with 3-hydroxy-3-indolyloxindoles.

The Zhang group described the first chiral imidodiphosphoric acid-catalyzed enantioselective Friedel–Crafts reactions of indoles and pyrroles with 3-hydroxy-3-indolyloxindoles, giving the 3,3-diaryloxindoles in excellent yields (99% yield) and with excellent enantioselectivities (98% ee) at low catalyst loading (Scheme 51) [69]. The protocol developed was also suitable for a gram scale synthesis, thus shows promising in the constrction of analogs of natural product trisindoline for further biological evaluation. In this work, the authors obtained diindolyloxindoles, in which the oxindole motif was attached to the 3-position of indoles, while Shi et al. reported that they obtained regioisomers when 3-methylindoles were used (Scheme 49) [66], indicating that the 3-methyl group was important for the observed regioselectivity.

**Scheme 52:**
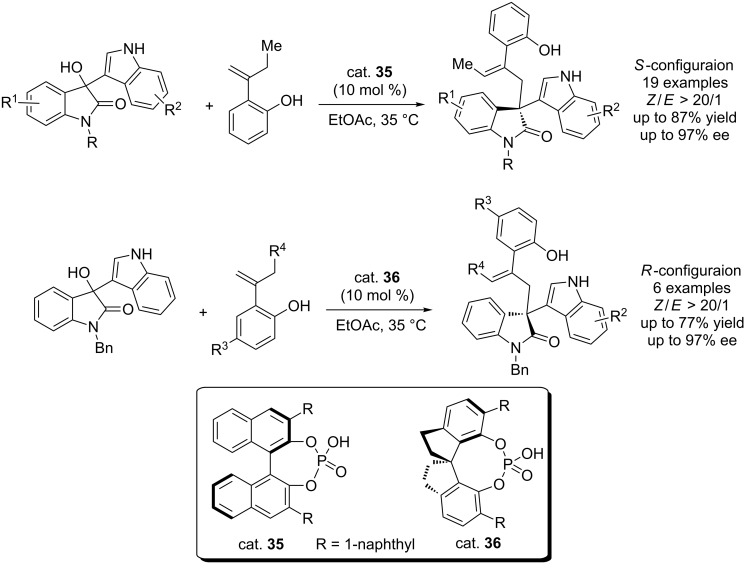
CPA-catalyzed enantioselective allylation of 3-indolylmethanols.

Shi et al. established a chiral CPA-catalyzed asymmetric allylation of 3-indolylmethanols through the hydrogen bond activating mode, which incorporated the 3-hydroxy-3-indolyloxindoles and *o*-hydroxystyrenes into the 3-allyl-3-indolyloxindoles, featuring one all-carbon stereogenic center and a (*Z*)-C=C bond. All products were obtained in excellent enantioselectivity (up to 97% ee) and (*Z*)-selectivity (up to >20/1 *Z*/*E* ratio, Scheme 52) [70]. Besides, the absolute configuration of the products can be controlled by the chiral CPA, selectively affording the (*R*)- or (*S*)-enantiomer based on the catalyst used.

**Scheme 53:**
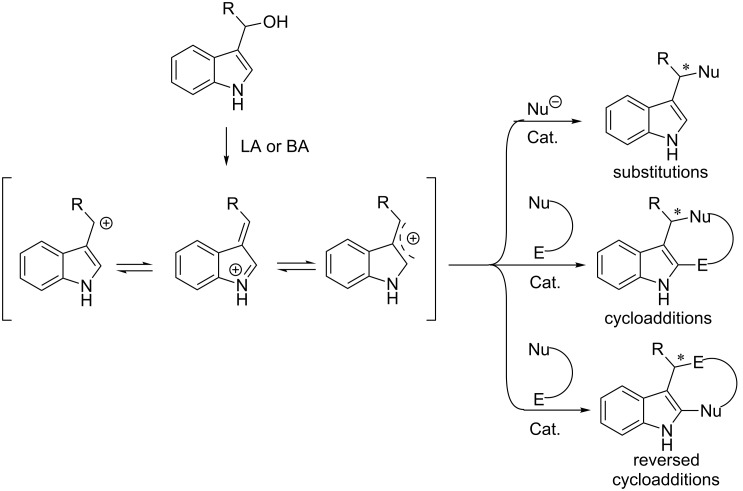
3-Indolylmethanol-based substitution and cycloaddition reactions.

3-Indolylmethanols have been recongnized as reactive precursors in organic synthesis for their characteristics of convertion to vinyliminium species or the delocalized carbocation intermediates in the presence of Lewis or Brønsted acids (LA or BA). In addition to the aforementioned studies on the nucleophilic substitutions, another research focus is the 3-indolylmethanol-based cycloaddition reactions for constructing structurally novel and complex polycyclic scaffolds (Scheme 53).

**Scheme 54:**
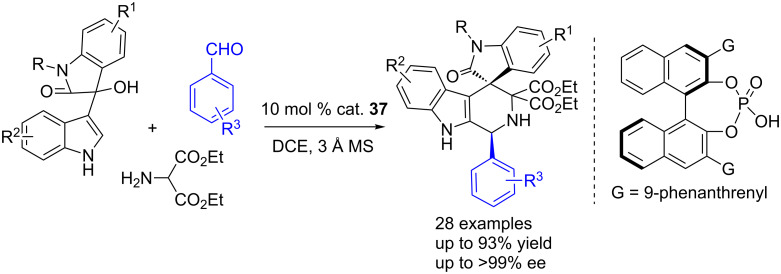
CPA-catalyzed asymmetric [3 + 3] cycloaddtion reactions of 3-indolylmethanols with azomethine ylides.

A pioneering work has been done on the 3-indolylmethanol-involved cycloaddtion reactions by the Shi group. In 2014, Shi et al. developed a CPA-catalyzed asymmetric [3 + 3] cycloaddition of 3-indolylmethanols with azomethine ylides generated in situ from the corresponding amines and aldehydes, affording the piperidine-fused spirooxindole frameworks in high yields (up to 93% yield) and with excellent enantioselectivities (>99% ee), albeit with moderate diastereoselectivities (Scheme 54) [71].

**Scheme 55:**
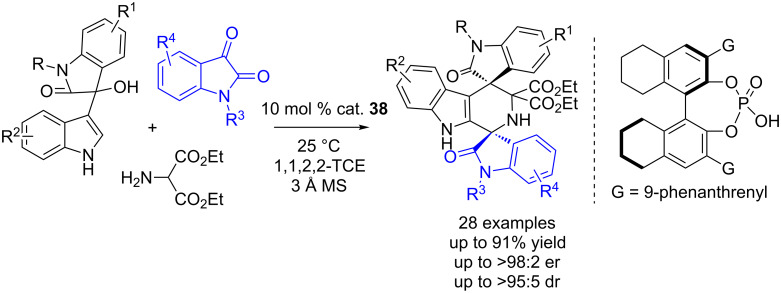
CPA-catalyzed three-component cascade Michael/Pictet–Spengler reactions of 3-indolylmethanols and azomethine ylides.

Similarly, the same group developed another CPA-catalyzed three-component cascade Michael/Pictet–Spengler reaction of 3-indolylmethanols and azomethine ylides, in which isatins were used in place of aldehydes (Scheme 55) [72]. Based on this method, they obtained structurally novel and complex bisspirooxindoles in excellent enantioselectivities (>95:5 dr, up to 98:1 er).

**Scheme 56:**
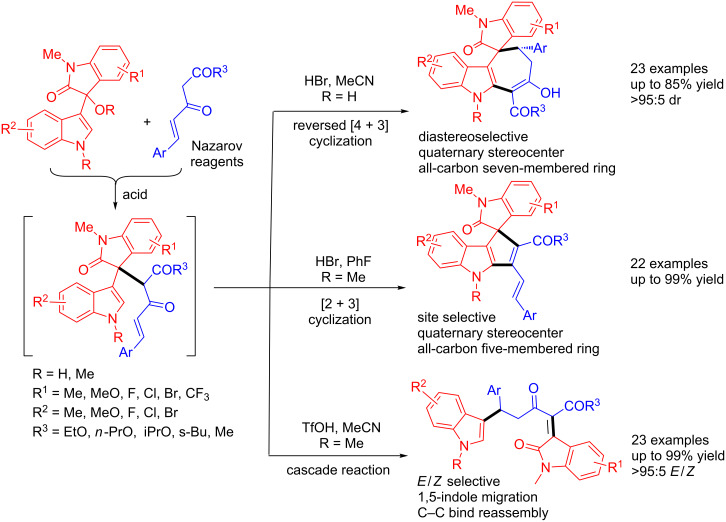
Acid-promoted chemodivergent and stereoselective synthesis of diverse indole derivatives.

Very recently, Shi et al. revealed an unprecedented acid-promoted intermediate-dependent unusual [4 + 3], [3 + 2] and cascade reactions of 3-indolylmethanols with Nazarov reagents, leading to the construction of diverse indole derivatives (Scheme 56) [73]. 3-Indolylmethanols with Nazarov reagents reacted smoothly in the presence of HBr in MeCN, affording the cyclohepta[*b*]indole frameworks through an electronically reversed [4 + 3] cyclization, while when treated with HBr in PhF, cyclopenta[*b*]indole skeletons were accessed through an site-selective [3 + 2] cyclization. Differently, treatment of 3-indolylmethanols with Nazarov reagents with TfOH in MeCN led to the generation of indoles through a cascade reaction in an (*E*/*Z)*-selective mode. These unusual reactions may serve as stereoselective and chemodivergent methods for accessing indole-containing scaffolds.

**Scheme 57:**
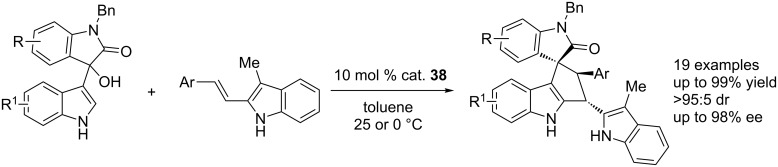
CPA-catalyzed asymmetric formal [3 + 2] cycloadditions.

Shi and co-workers also established a CPA (cat. **38**)-catalyzed asymmetrc [3 + 2] cycloaddition of 3-indolylmethanols with 3-methyl-2-vinylindoles, affording spiro[cyclopenta[*b*]indole-1,3’-oxindole] scaffolds in moderate to good yields (72–99% yield) and with excellent diastereoselectivities (>95:5 dr) and enantioselectivities (90–98% ee, Scheme 57) [74].

**Scheme 58:**
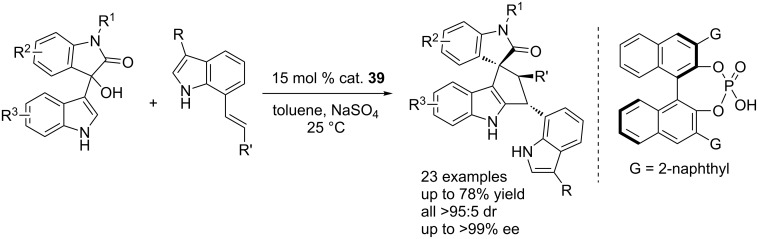
CPA-catalyzed enantioselective cascade reactions for the synthesis of C7-functionlized indoles.

Following above success, Shi et al. developed other CPA-catalyzed cascade reactions of 3-indolylmethanols with 7-vinylindoles, affording C7-functionalized indoles and constructing the cyclopenta[*b*]indole and spirooxindole frameworks with excellent diastereoselectivities (all >95:5 dr) and enantioselectivities (up to >99% ee, Scheme 58) [75]. Mechanistic studies showed that this cascade reaction proceeded via a vinylogous Michael addition/Friedel–Crafts process involving a dual H-bonding activation of substrates.

**Scheme 59:**
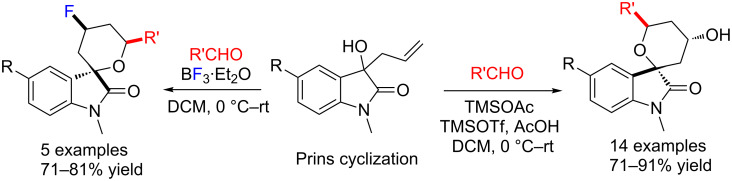
Lewis acid-promoted Prins cyclization of 3-allyl-3-hydroxyoxindoles with aldehydes.

In 2015, Wang and co-workers reported Lewis acid-promoted Prins cyclization reactions of 3-allyl-3-hydroxyoxindoles with aldehydes, accessing functionalized spirooxindole pyrans as single diastereoisomers in good yields (71–91%) under mild conditions [76]. Interestingly, when BF_3_·Et_2_O was used, they obtained the biologically important fluorinated spirooxindoles in good yields (71–81%, Scheme 59).

**Scheme 60:**
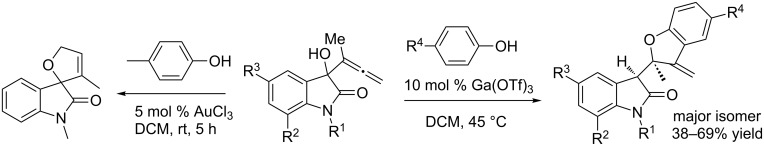
Ga(OTf)_3_-catalyzed reactions of allenols and phenols.

In the same year, Alcaide et al. described the Ga(OTf)_3_-catalyzed cyclization reactions of allenols with phenols under mild conditions, offering the dihydrobenzofuran-appended oxindoles through the addition/cyclization sequence (Scheme 60) [77]. After screening Lewis acid catalysts, they found that Ga(OTf)_3_ was the best catalyst to promote this kind of reactions. However, the major isomers were obtained in moderate yields (38–69%) and with poor diastereoselectivities. For *N*-unsubstituted 3-hydroxyoxindoles (R^1^ = H) and phenols with bulky substitutents, the corresponding products were found to have improved diastereoselectivities. Phenols with electron-withdrawing groups failed to afford the expected products under the same conditions. Mechanistically, Ga(OTf)_3_ may activate the allene and phenol substrates simultaneously via the bidentate model. In contrast, the spirocyclic 2,5-dihydrofuran was obtained in 98% yield when the π acid AuCl_3_ (5 mol %) was used. AuCl_3_ may coordinate one of the allene double bonds via a monodentate model. The phenol substrate did not participate in this reaction.

**Scheme 61:**
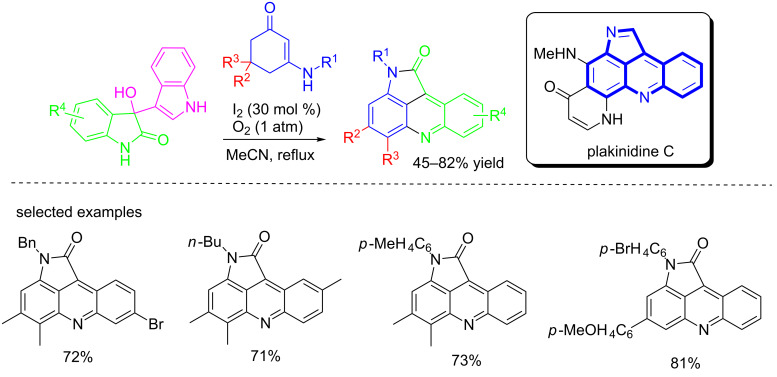
I_2_-catalyzed construction of pyrrolo[2.3.4-*kl*]acridines from enaminones and 3-indolyl-3-hydroxyoxindoles.

In 2013, Ji and co-workers described the I_2_-catalyzed construction of pyrrolo[2,3,4-*kl*]acridines (the core structure of plakinidine C) from enaminones and 3-indolyl-3-hydroxyoxindoles through a cascade ring-opening/recyclization/methyl migration sequence (Scheme 61) [78]. The reactions proceeded well in MeCN in the presence of catalytic I_2_ (30 mol %) under an O_2_ atmosphere, giving the products in moderate to good yields (45–82%). Reactions performed in other solvents (EtOH, AcOH, toluene) gave the products in lower yields (<40%). Besides, the possible mechanism was also proposed based on the control experiments and previous reports. This protocol may provide an efficient entry to polycyclic scaffolds for further biological screening.

**Scheme 62:**
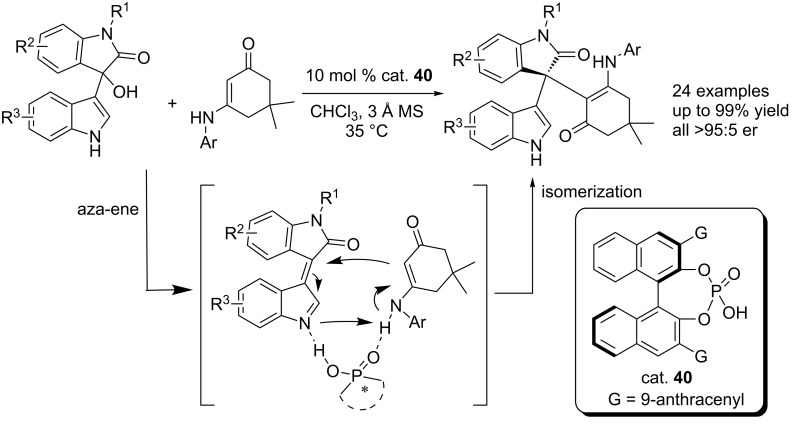
CPA-catalyzed asymmetric aza-ene reaction of 3-indolylmethanols with cyclic enaminones.

Similarly, Shi et al. established CPA-catalyzed highly enantioselective aza-ene reactions of cyclic enaminones with 3-indolyl-3-hydroxyoxindoles, incorporating enaminones and 3-indolyl-3-hydroxyoxindoles into C3-functionlized chiral indoles in high yields (up to 99% yield) and with excellent enantioselectivities (up to 95:5 er, Scheme 62) [79]. The NH group of the indoles was proved to be critically important for the reaction, for *N*-protected indole substrates, no reactions occurred under the same reaction conditions.

**Scheme 63:**
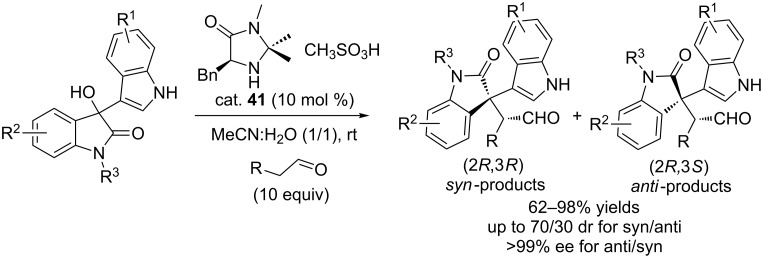
Asymmetric *α*-alkylation of aldehydes with 3-indolyl-3-hydroxyoxindoles.

In 2013, Li and co-workers described the asymmetric α-alkylation of aldehydes with 3-indolyl-3-hydroxyoxindoles in aqueous medium in the presence of catalytic MacMillan catalyst (10 mol %, cat. **41**), affording the desired products in good to excellent yields (62–98%) and with moderate diastereoselectivities (up to 70:30 dr for *syn*/*anti*) and excellent enantioselectivities (>99% ee for *anti*/*syn*-products, Scheme 63) [80]. A trace amount of the desired product was observed when the reaction was carried out in organic solvents (MeCN, DMSO, EtOAc, and DCM) and H_2_O. The mixture of MeCN and H_2_O (1:1) as the solvent was crucial for the reactions, although the starting materials were poorly solubilized. However, ten equivalents of aldehyde were used in the reactions.

**Scheme 64:**
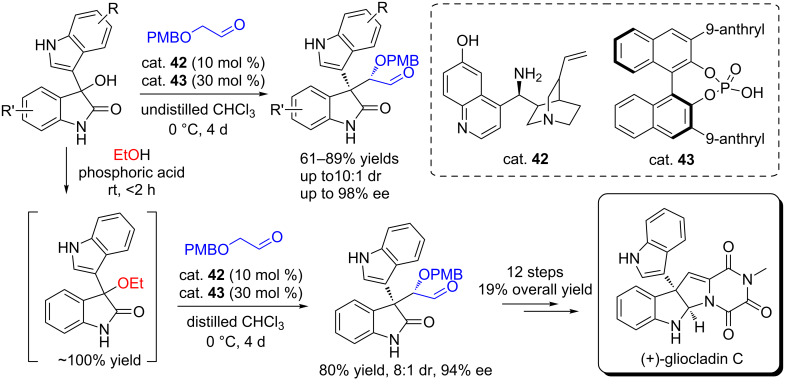
Organocatalytic asymmetric *α*-alkylation of enolizable aldehydes with 3-indolyl-3-hydroxyoxindoles and its application in the total synthesis of natural (+)-gliocladin C.

Similarly, Gong and co-workers reported an organocatalytic α-alkylation of enolizable aldehydes with 3-indolyl-3-hydroxyoxindoles co-catalyzed by the *cinchona* alkaloid amine (cat. **42**, 10 mol %) and CPA (cat. **43**, 30 mol %), affording the desired products in moderate to good yields (61–89% yields) and with good diastereoselectivities (up to 10/1 dr) and excellent enantioselectivities (up to 99% ee, Scheme 64) [81]. The poor yield, diastereoselectivity and enantioselectivity were observed when the reactions were performed only in the presence of 10 mol % *cinchona* alkaloid amine catalyst and TFA (30 mol %). It was found that (*S*)-BINOL-derived CPA catalyst can significantly improve the stereochemical control of the *cinchona* alkaloid amine catalyst, while (*R*)-CPA completely inhibited the catalytic activity. Interestingly, the authors found that the yields and enantioselectivities dramatically decreased when the reactions were performed in distilled CHCl_3_ (EtOH free) compared to the reactions carried out in undistilled commercial CHCl_3_ (containing a trace of EtOH as the stabilizer). Comparable results were obtained in distilled CHCl_3_ using the alcohol (MeOH, EtOH, and isopropanol) as the additive or using the 3-ethoxy-3-hydroxyoxindole as the substrate in distilled CHCl_3_ under the same conditions. They proposed that the alcohol affected the stereoselectivity possibly through controlling the generation rate of *trans/cis* vinylogous iminium intermediates in the dehydration step. The limitation is that the reactions need long reaction time (4 days). Based on this protocol, the authors successfully achieved the total synthesis of natural (+)-gliocladin C in 19% overall yield within 12 steps.

Additionally, 3-hydroxyoxindoles, as versatile precursors, have also found applications in the constrction of of biologically important 3-aminooxindoles [82–83], spirooxindoles [84], and disubstituted oxindoles [85].

## Conclusion

3-Hydroxyoxindole scaffolds are prevalent in natural products and biologically relevant molecules, and have exhibited diverse biological activities such as inhibition of proteasome, antagonizing GHSR and inhibiting growth of human cancer cells. Interestingly, for some 3-hydroxyoxindoles (e.g., YK-4-279 in Figure 1), different enantiomers display a remarkably different activity. Inspired by the structural features and biological potential of 3-hydroxyoxindoles, many efforts have been devoted to developing asymmetric strategies toward the construction of chiral 3-hydroxyoxindoles including transition metal catalysis and organocatalysis. Various transition metals (in combination with appropriate chiral ligands) and organocatalysts have proven to be efficient in catalyzing different kinds of reactions for constructing chiral 3-hydroxyoxindoles in good to excellent yields and with good to excellent enantioselectivities as well as diastereoselectivities. Transition metal-catalyzed allylation, Friedel–Carfts reactions, and the asymmetric aldol reactions are the most employed strategies toward the construction of 3-substituted 3-hydroxyoxindoles in high yields and with excellent enantioselectivities. Ir-catalyzed asymmetric intramolecular hydroarylation of α-ketoamides serves as an alternative way to build the 3-hydroxyoxindole core with diverse substituents. Additionally, 3-hydroxyoxindoles, especially the 3-indolyl-3-hydroxyoxindoels as versatile intermediates have been used for the total synthesis of natural products (e.g., (+)-gliocladin C) and constructing structurally complex and biologically interesting polycyclic heterocycles and biologically privileged spirooxindole scaffolds. In the presence of Lewis acids, 3-hydroxyoxindoles can be converted to iminium intermediates through dehydration, which then can react with electron-rich aromatics or nucleophiles to form 3,3-disubstituted oxindoles. This transformation could be potentially used for accessing novel scaffolds in drug discovery pursuits. Given the importance of 3-substituted 3-hydroxyoxindoles in organic synthesis and the identification of drug leads or probes, more efficient strategies based on transition metal catalysis and organocatalysis will be pursued. On the other hand, 3-hydroxyoxindoles, followed by installation of functional groups on the hydroxy group could be used for constructing biologically interesting and structurally novel scaffolds, such as 3,3-disubstituted oxindoles and privileged spirooxindoles, which are prevalent in natural products and pharmaceutical agents (e.g., CFI-400945, SAR405838, KAE609). Compared to other oxindole compounds, bioactivies of 3-hydroxyoxindoles are still relatively less studied. We believe that more asymmetric strategies will be reported in the near future, and 3-hydroxyoxindoles, as promising but less studied synthetic intermediates, will find more applications in the total synthesis of natural products and small-molecule libratry construction for new drug screening.
